# The Lrat^−/−^ Rat: CRISPR/Cas9 Construction and Phenotyping of a New Animal Model for Retinitis Pigmentosa

**DOI:** 10.3390/ijms22137234

**Published:** 2021-07-05

**Authors:** Céline Koster, Koen T. van den Hurk, Colby F. Lewallen, Mays Talib, Jacoline B. ten Brink, Camiel J. F. Boon, Arthur A. Bergen

**Affiliations:** 1 Department of Human Genetics Amsterdam, Section of Ophthalmogenetics, Amsterdam University Medical Centers (AUMC), University of Amsterdam (UvA), Location Meibergdreef, 1105 AZ Amsterdam, The Netherlands; c.koster@amsterdamumc.nl (C.K.); k.t.vandenhurk@amsterdamumc.nl (K.T.v.d.H.); j.b.tenbrink@amsterdamumc.nl (J.B.t.B.); 2 Georgia Institute of Technology, G.W. Woodruff School of Mechanical Engineering, Atlanta, GA 30313, USA; colby.lewallen@gatech.edu; 3 Department of Ophthalmology, Leiden University Medical Center, 2333 ZA Leiden, The Netherlands; m.talib@lumc.nl (M.T.); Camiel.boon@amsterdamumc.nl (C.J.F.B.); 4 Department of Ophthalmology, Amsterdam University Medical Centers (AUMC), University of Amsterdam (UvA), Location Meibergdreef, 1105 AZ Amsterdam, The Netherlands; 5 The Netherlands Institute for Neuroscience (NIN-KNAW), 1105 BA Amsterdam, The Netherlands

**Keywords:** lecithin retinol acyltransferase (LRAT), pigmented rat model, retinal pigment epithelium (RPE), retinal degeneration, retinal dystrophy, retinitis pigmentosa

## Abstract

Purpose: We developed and phenotyped a pigmented knockout rat model for lecithin retinol acyltransferase (LRAT) using CRISPR/Cas9. The introduced mutation (c.12delA) is based on a patient group harboring a homologous homozygous frameshift mutation in the *LRAT* gene (c.12delC), causing a dysfunctional visual (retinoid) cycle. Methods: The introduced mutation was confirmed by DNA and RNA sequencing. The expression of *Lrat* was determined on both the RNA and protein level in wildtype and knockout animals using RT-PCR and immunohistochemistry. The retinal structure and function, as well as the visual behavior of the *Lrat*^−/−^ and control rats, were characterized using scanning laser ophthalmoscopy (SLO), optical coherence tomography (OCT), electroretinography (ERG) and vision-based behavioral assays. Results: Wildtype animals had high *Lrat* mRNA expression in multiple tissues, including the eye and liver. In contrast, hardly any expression was detected in *Lrat*^−/−^ animals. LRAT protein was abundantly present in wildtype animals and absent in *Lrat*^−/−^ animals. *Lrat*^−/−^ animals showed progressively reduced ERG potentials compared to wildtype controls from two weeks of age onwards. Vison-based behavioral assays confirmed reduced vision. Structural abnormalities, such as overall retinal thinning, were observed in *Lrat*^−/−^ animals. The retinal thickness in knockout rats was decreased to roughly 80% by four months of age. No functional or structural differences were observed between wildtype and heterozygote animals. Conclusions: Our *Lrat*^−/−^ rat is a new animal model for retinal dystrophy, especially for the *LRAT*-subtype of early-onset retinal dystrophies. This model has advantages over the existing mouse models and the RCS rat strain and can be used for translational studies of retinal dystrophies.

## 1. Introduction

**Figure 1:**
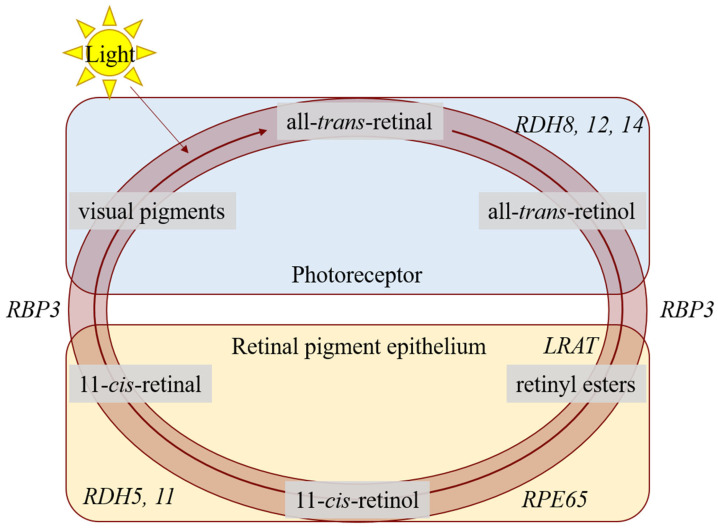
A schematic overview of the visual cycle in the photoreceptors (PRs) and the retinal pigment epithelium (RPE). In the PRs, 11-*cis*-retinal couples to an opsin protein, forming rhodopsin. Upon activation by photons, 11-*cis*-retinal is isomerized to all-*trans*-retinal. The retinol dehydrogenases (encoded by *RDH8*, *RDH12*, *RDH14*) reduce all-*trans*-retinal to all-*trans*-retinol, and this metabolite is moved to the RPE by retinoid-binding protein (encoded by *RBP3*). In the RPE, it is esterified by lecithin:retinol acyltransferase (encoded by *LRAT*), after which it is converted to 11-*cis*-retinol by retinal pigment epithelium-specific 65 kDa protein (encoded by *RPE65*). Retinol dehydrogenases (encoded by *RDH5* and *RDH11*) convert 11-*cis*-retinol to 11-*cis*-retinal, and retinoid-binding protein moves it back to the PR. For further explanation, see the text.

Retinitis pigmentosa (RP), Leber congenital amaurosis (LCA), and retinitis punctata albescens (RPA) are severe early-onset retinal dystrophies that cause visual impairment, nystagmus, progressive nyctalopia, and finally, blindness. This heterogeneous retinal dystrophy disease group is characterized by damage to the retinal pigment epithelium (RPE)–photoreceptor (PR) complex. This results usually in progressive dysfunction of the rod photoreceptor cells, often followed by progressive cone degeneration. RP, LCA, and RPA are caused by mutations in virtually all genes encoding proteins acting in the retinoid cycle [1,2,3,4]. Indeed, for normal vision, a functionally valid retinoid cycle is essential: In the healthy situation, vitamin A (retinol) is the primary substrate for several functional retinoids’ biosynthesis in the retinoid cycle. Then, the vitamin A-derivatives are shuttled from the RPE to the PRs. There, opsins are light-activated and the visual pigments transform the light energy in a cellular signal, initiating the visual cascade and resulting in a physiological response in the PR cell. After light activation, the cycle regenerates the visual pigments that are used after light activation of rhodopsin (see Figure 1). Upon photoactivation, a configurational change of the visual pigment 11-*cis*-retinal to all-*trans*-retinal is induced in the PR cells’ outer segments. Subsequently, all-*trans*-retinal is reduced to all-*trans*-retinol and diffuses from the PRs back to to the RPE cells. In the RPE, all-*trans*-retinol is esterified to all-*trans*-retinyl-ester by the enzyme lecithin:retinol acetyltransferase (LRAT), after which all-*trans*-retinyl-ester is subsequently the substrate for the enzyme retinal pigment epithelium-specific protein 65 kDa (RPE65). RPE65 converts all-*trans*-retinyl-ester to 11-*cis*-retinol, after which 11-*cis*-retinol is oxidized by retinol dehydrogenase (RDH) enzymes to 11-*cis*-retinal. Finally, to complete the cycle, 11-*cis*-retinal is shuttled back to the PRs, where it can be used for a new round of phototransduction.

**Figure 2:**
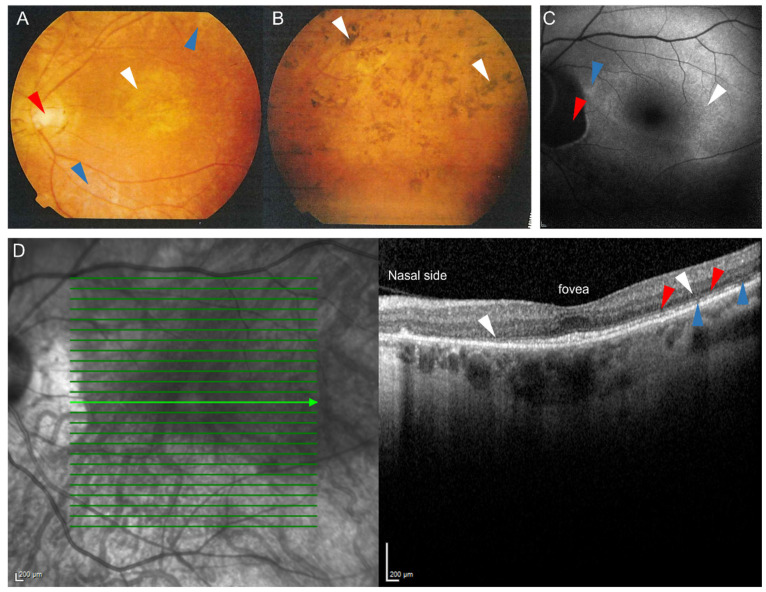
Representative fundus photographs (**A**,**B**), fundus autofluorescence image (**C**) and an optical coherence tomography (OCT) scan (**D**) of patients carrying the c.12delC mutation in *LRAT* suffering from *LRAT*-associated retinal dystrophy. Fundus photograph of the central (**A**) and peripheral area (**B**) of the left eye of a 39-year old patient. (**A**): Atrophic alterations of the retinal pigment epithelium in the macula (white arrow) and around the vascular arcades (blue arrows), along with retinal atrophy around the optic disc, which shows some temporal pallor (red arrow) are visible. The vessels are attenuated. (**B**): The peripheral retina showed retinal atrophy and bone-spicule-like hyperpigmentation. (**C**): Fundus autofluorescence image of a 57-year old patient, showing a subtle hyperautofluorescent ring around a relatively preserved central macula (white arrow) and a juxtapapillary patch of absent autofluorescence (red arrow), sharply outlined by a hyperautofluorescent border. The inferior posterior pole shows granular hypo-autofluorescence (blue arrow), indicating more atrophy of the retinal pigment epithelium in the area outside of the hyperautofluorescent ring. (**D**): Spectral-domain OCT (SD-OCT) scan of a 57-year old patient, showing relative preservation of the outer nuclear layers, the external limiting membrane, and the ellipsoid zone at the level of the fovea. In the parafovea and perifovea, thinning of the outer nuclear layer is seen (white arrows), along with interruptions of the external limiting membrane (red arrows) and the ellipsoid zone (blue arrows). These interruptions, along with the outer nuclear layer thinning, increase towards the peripheral macula and are more profound on the nasal side.

Thus, LRAT as well as RPE65 are essential for the regeneration of functional visual pigment in the part of the retinoid cycle that takes place in the RPE. A defect in either enzyme leads to an impaired retinoid cycle [5,6]. Indeed, mutations in the *RPE65* gene have been implicated in 6–8% of all LCA cases and up to 5% of childhood-onset RP [2,7]. *RPE65*-associated retinal disorders are the first for which human gene therapy has become available [8,9], with many other retinal dystrophies, including *LRAT*-associated retinal disorders, to follow [10]. Mutations in the *LRAT* gene are rare and cause LCA, childhood-onset RP, and RPA-/fundus albipunctatus-like phenotypes in <1% [2,3,7,11,12,13] (Figure 2). Interestingly, considerable phenotypic variability has been described in association with *LRAT* mutations [13].

The rat *Lrat* gene was characterized in 1999 by Ruiz and colleagues [14]. They described that the *Lrat* gene is highly expressed in several tissues, including the RPE in the eye, liver, heart, lung, skeletal muscle, skin, mammary tissue, testis, intestine, adrenal gland, and pancreas [14,15]. Multiple transcripts in a size range of 1.5–5 kb were identified in various human tissues, rodent tissues and cell lines. However, the translated protein from both the long and shorter transcripts contains 230 amino acids and is 25.8 kDa in size [14,15,16,17]. This demonstrates that the long 3’UTR after the ORF in the long transcripts did not affect the translation [17]. The widespread expression suggests a role for LRAT in several biological processes in multiple organs. However, patients with *LRAT* mutations only have retinal dystrophy and do not have other obvious pathogenic systemic abnormalities. The reason for this is not clear, but it suggests that the regulation of *LRAT* mRNA expression is complex and yet to be fully elucidated.

The c.12delC (NM_004744.4) mutation in the *LRAT* gene segregated perfectly in families with RPA, as shown by Littink and colleagues [4]. The mutation causes a frameshift and a premature stop codon 53 amino acids downstream (p.M5CfsX53), theoretically resulting in a truncated protein. These patients suffer from nyctalopia, decreased non-recordable scotopic electroretinography (ERG) measurements, and an overall decrease in visual sensitivity and acuity. Talib and colleagues examined patients with this homozygous mutation in the *LRAT* gene in a long-term follow-up study. Although the disease’s progression was variable and slow, the follow-up showed that complete blindness was generally reached between 50 and 60 years of age [13]. The first symptoms started to show within the first decade of life in all patients, with nyctalopia being usually the first symptom within the first year of life. The clinical course of *LRAT*-associated phenotypes appears similar to the clinical course in patients with *RPE65* mutations [13].

The lack of human representative animal models severely hampers the development of effective treatments for retinal degenerative diseases. *Rpe65* and *Lrat* deficient mice have previously been used to study their RP-related phenotype and test potential experimental treatments’ efficacies [5,6,18]. These strains show that the rods degenerate slowly, and the cones degenerate rapidly, with eventual complete loss of cone function [19]. Additionally, both strains had severely decreased to nearly absent ERG responses [5,6,18]. In these mouse strains, supplementation of visual cycle metabolites, such as retinoids [20] and 9-cis-retinyl acetate (QLT091001), seem to be efficacious to maintain the ERG responses as long as there is sufficient retinal integrity to support functional improvement [21,22]. Additionally, gene replacement therapy using adeno-associated viruses (AAVs) or lentiviruses have been used in *Rpe65*^−/−^ [23,24,25,26,27] and *Lrat*^−/−^ mice [22], resulting in the maintenance of retinal integrity and the improvement of ERG a- and b-wave amplitudes.

Experimental gene therapy is generally performed by careful intravitreal or subretinal injections in relavant host mice. However, mouse eyes are extremely small, hampering the effectiveness of these techniques. Furthermore, ocular surgery in mouse eyes, such as placing a sheet of RPE cells into the subretinal space, using commercially available devices, is nearly impossible. Consequently, bigger rat eyes are preferable for these types of experimental therapies. Their larger eye volume enables easier access for injections and makes work with commercially available surgical tools possible [28,29]. Despite this seeming advantage, only a limited number of genetic rat strains with inherited RD are attainable at the time of writing. The Royal College of Surgeons (RCS) is a spontaneous genetic rat strain with inherited RD, and this model is currently widely used for testing the efficiency of therapies for RDs [30,31,32]. The strain harbors a mutation in the *Mertk* gene, which causes the RPE to fail to phagocytose the shed photoreceptor outer segments. Despite the apparent advantages of a rat model with larger eyes, photoreceptor debris in the RCS rat accumulates in the subretinal space, initiating spontaneous retinal degeneration and hampering experimental treatment modalities [33].

The work presented here entails the CRISPR/Cas9 mediated construction and characterization of a new *Lrat* deficient Brown Norway rat strain. Visual examination and follow-up of the knockout rat showed that it is a human-representative animal model for RP and, more specifically, RPA. The mutation (c.12delA), which we introduced, is based on a known patient group previously described by our group [4,13]. This new rat model could be used to develop potential (experimental) therapies to treat this specific patient group and patients harboring similar genotypes and/or phenotypes.

## 2. Results

### 2.1. Generation of Lrat Knockout (KO) Rats

**Figure 3:**
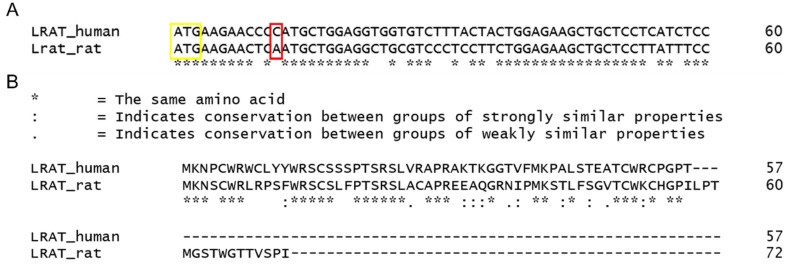
Alignment of the human and rat genomic DNA and protein sequences. (**A**): Alignment of the first 60 nucleotides of the *LRAT* and *Lrat* ORF starting with the start codon (indicated by the yellow box). The red box indicates the nucleotide that is deleted in patients (c.12delC) and the rat equivalent (c.12delA). (**B**): Alignment of the predicted resulting peptide in patients and rats harboring the deletion (c.12del). The single nucleotide deletion in the genomic DNA causes a frameshift from the fifth amino acid onwards and a truncated protein in both humans (57 amino acids) and rats (72 amino acids).

The *Lrat* mutation of interest was selected based on a mutation (c.12delC) occurring in a patient group’s DNA within our hospital [4,13]. Bio-informatic homology analyses between human and rat genomic DNA sequences revealed that the rat’s targeted mutation is c.12delA (Figure 3A). The alignments of the predicted peptides in patients and rats harbouring the deletion (c.12del) are shown in Figure 3B. For the complete alignments of human and rat mRNA and amino acid sequences, see Supplementary File 1.

**Figure 4:**
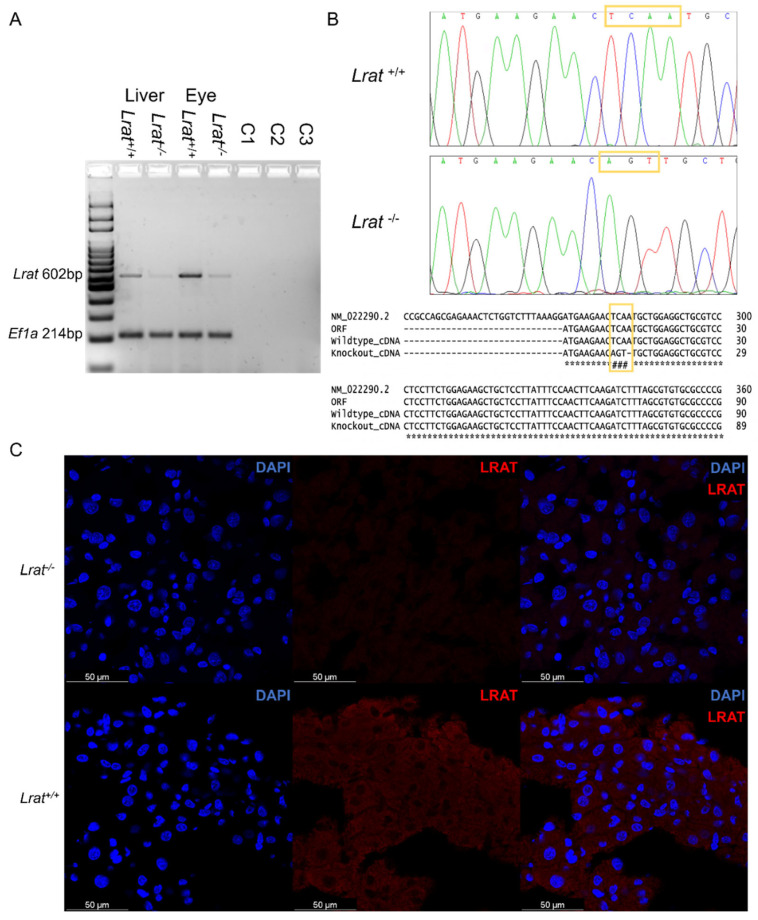
(**A**): mRNA expression of *Lrat* and *Ef1a* shown for *Lrat*^+/+^ and *Lrat*^−/−^ animals for liver and eye tissues. *Lrat* expression was found in both wildtype liver and eye tissues. Significantly less RNA is found in *Lrat*^−/−^ tissues. C1–3: Negative controls −/−cDNA in PCR reaction, −/−RNA in cDNA synthesis, and −/−SuperScriptIII in cDNA synthesis. (**B**): the presence of the introduced single nucleotide deletion and silent mutation in the *Lrat* mRNA of the Brown Norway rat was confirmed by Sanger sequencing. The rat reference sequence was retrieved from the NCBI database, Accession NM_02290.2. The open reading frame (ORF) of *Lrat* was determined using the ORFfinder (NCBI). (**C**): LRAT protein presence is confirmed in the liver of *Lrat*^+/+^ animals and (almost completely) absent in *Lrat*^−/−^ liver tissue.

Of 18 pups born after CRISPR/Cas9 editing, 4 carried mutations at the predicted 5’ and/or 3’ guide RNA target site, as judged from surveyor assays. The mutation of interest (c.12delA) was detected in two founders by sequencing a PCR product surrounding the predicted mutation (see Figure 4B). The DNA sequences’ alignment in these two founders revealed that a local insertion and deletion were introduced in the Brown Norway rat’s genome. Four nucleotides were deleted (c.10–13: TCAA), and three nucleotides were inserted at that spot (AGT), resulting in a silent mutation at p.4Ser and a frameshift afterward due to the targeted single nucleotide deletion (c.12delA). We found that the introduced mutation cosegregated with the disease phenotype through multiple generations without observing potential recombinations, which strongly suggests the introduced mutation’s pathogenicity.

Intercrosses of heterozygotes (*Lrat*^+/−^) rats produced homozygous *Lrat*^−/−^ progeny in standard Mendelian ratios (see Supplementary Table S2). Homozygous (*Lrat*^−/−^) crossings produced normal-sized litters, averaging 8 pups (6–12, median 8). *Lrat*^−/−^ rats survived for over one year. No spontaneous phenotype was observed judging from their social behavior and health parameters (e.g., weight progression, see Supplementary Figure S1) under normal laboratory circumstances. This indicates that *Lrat*^−/−^ rats have no readily apparent disease phenotype, as can be judged from standard laboratory observations.

### 2.2. Lrat Gene and Protein Expression in Rat Tissues of Wildtype, Heterozygous, and Lrat Knockout (KO) Rats

We analyzed the presence of *Lrat* mRNA using PCR in the liver and eye tissues of *Lrat*^+/+^ and *Lrat*^−/−^ animals. We found a high abundance of *Lrat* transcript in both tissues of the *Lrat*^+/+^ animals (Figure 4A). Clearly, less *Lrat* transcript in *Lrat*^−/−^ was observed. This observation was confirmed using a second set of RT-PCR primers. Other tissues of *Lrat*^−/−^, *Lrat*^+/−^, and *Lrat*^+/+^ rats, including the kidneys, spleen, small intestine, lung, brain, and testis, were also tested for *Lrat* transcript presence, see Supplementary Figure S2. We found *Lrat* transcript to be abundant in the eyes, liver and testis. Less expression was found in lung tissue. After finding minimal *Lrat* mRNA in homozygous knockout tissues, we confirmed the single nucleotide deletion in the cDNA produced from whole mRNA samples of multiple animals. The sequences are shown in Figure 4B, including the alignment to the NCBI’s reference sequence and our wildtype Brown Norway animals. In agreement with the mRNA expression, we observed, using immunofluorescence, that LRAT protein was abundantly present in liver sections of *Lrat*^+/+^ animals and (almost completely) absent in liver sections of *Lrat*^−/−^ animals (Figure 4C).

### 2.3. In Vivo Imaging of Retinal Structures Using Non-Invasive Scanning Laser Ophthalmoscopy (SLO) and Optical Coherence Tomography (OCT)

**Figure 5:**
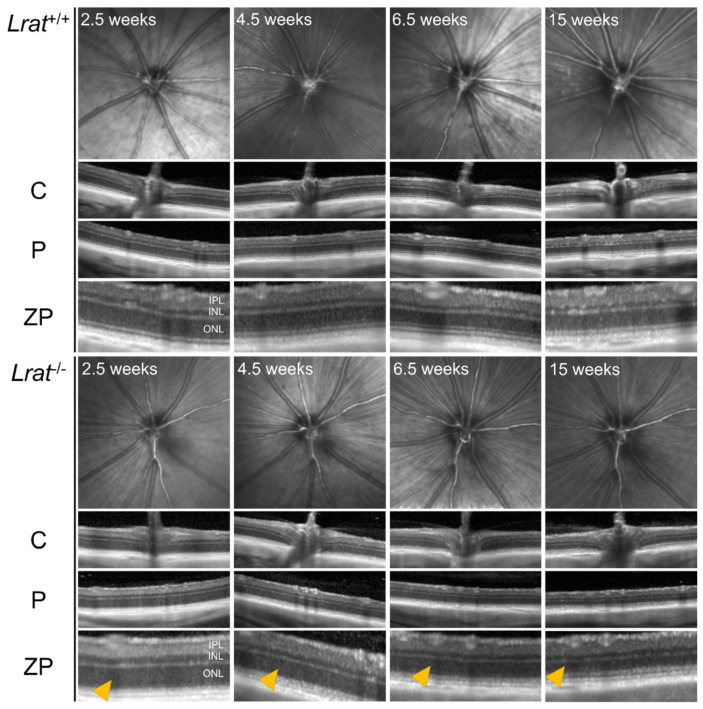
Representative qualitative SLO and OCT images of *Lrat*^+/+^ and *Lrat*^−/−^ rats at different ages. OCT scans from the same eye of the central area (C), including the optic disc and the peripheral area (P), are included for each genotype. Additionally, a more detailed image of the peripheral area is shown (ZP). It is possible to identify all retinal layers, including the inner plexiform layer (IPL), the inner nuclear layer (INL), and the outer nuclear layer (ONL) in both the wildtype and knockout animals from the OCT images. The layering of the retina becomes less distinct in the knockout animals over time. The retina of knockout animals degenerates over time and becomes thinner at later stages. Especially the ONL layer, representing the cell bodies of the PRs, becomes thinner over time (yellow arrows). For the thickness quantification, see Figure 6. No differences can be observed between wildtype and knockout SLO images.

**Figure 6:**
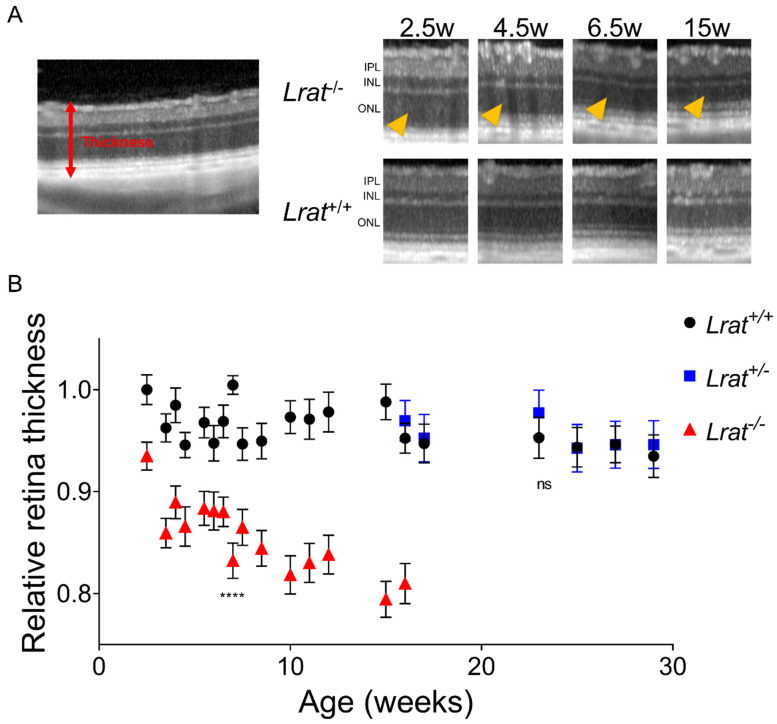
(**A**) quantitative representation of total retina thickness (**A**) at different ages of *Lrat*^+/+^ (black circles), *Lrat*^+/−^ (blue squares), and *Lrat*^−/−^ (red triangles) animals. The left panel of A shows how the total thickness was determined. The chosen scans for determining total thickness were the crossing of the optic nerve (ON = 0), the middle superior section (ON + 10), the superior section (ON + 20), the middle inferior section (ON − 10), and the inferior section (ON − 20). Within each selected OCT scan, retina thickness was determined on 1 mm intervals, with a 0.5 mm minimum distance from the optic nerve. All values were normalized to the wildtype retina’s thickness at 2 weeks of age and plotted with the standard error (panel (**B**)). Some variation between OCT measurements can be observed over time, although not significant within the wildtype and heterozygote animals. The retina of knockout animals degenerates over time and is significantly thinner than the retina of wildtype and heterozygous animals. The thinning is mostly due to the degeneration of the outer nuclear layer (ONL) (yellow arrows) (A). No significant differences are observed between wildtype and heterozygous animals. ns = non-significant; **** = *p* < 0.0001. IPL: inner plexiform layer; INL: inner nuclear layer.

We assessed the retinal morphology of *Lrat*^−/−^ rats and compared it to the retinal morphology of *Lrat*^+/+^ and *Lrat*^+/−^ rats over a timespan of 4 months with weekly intervals using non-invasive SLO and OCT. Representative fundus images and OCT scans of *Lrat*^+/+^ and *Lrat*^−/−^ animals of different ages (2.5, 4.5, 6.5, and 15 weeks of age) are presented in Figure 5. We observed an overall quantitative progressive degradation and significant thinning of the retina for *Lrat*^−/−^ animals overtime, as judged by OCT images (Figure 6B). The thickness decrease is mostly due to the outer nuclear layer’s degeneration, reflecting the loss of the PRs. At the same time, the inner plexiform layer and inner nuclear layer were similar to the wildtype controls. No apparent differences between the SLO and OCT images of *Lrat*^+/+^ and *Lrat*^+/−^ animals were seen (Supplementary Figure S3 and Figure 6B).

### 2.4. In Vivo Assessment of the Retinal Function Using Electroretinography (ERG)

**Figure 7:**
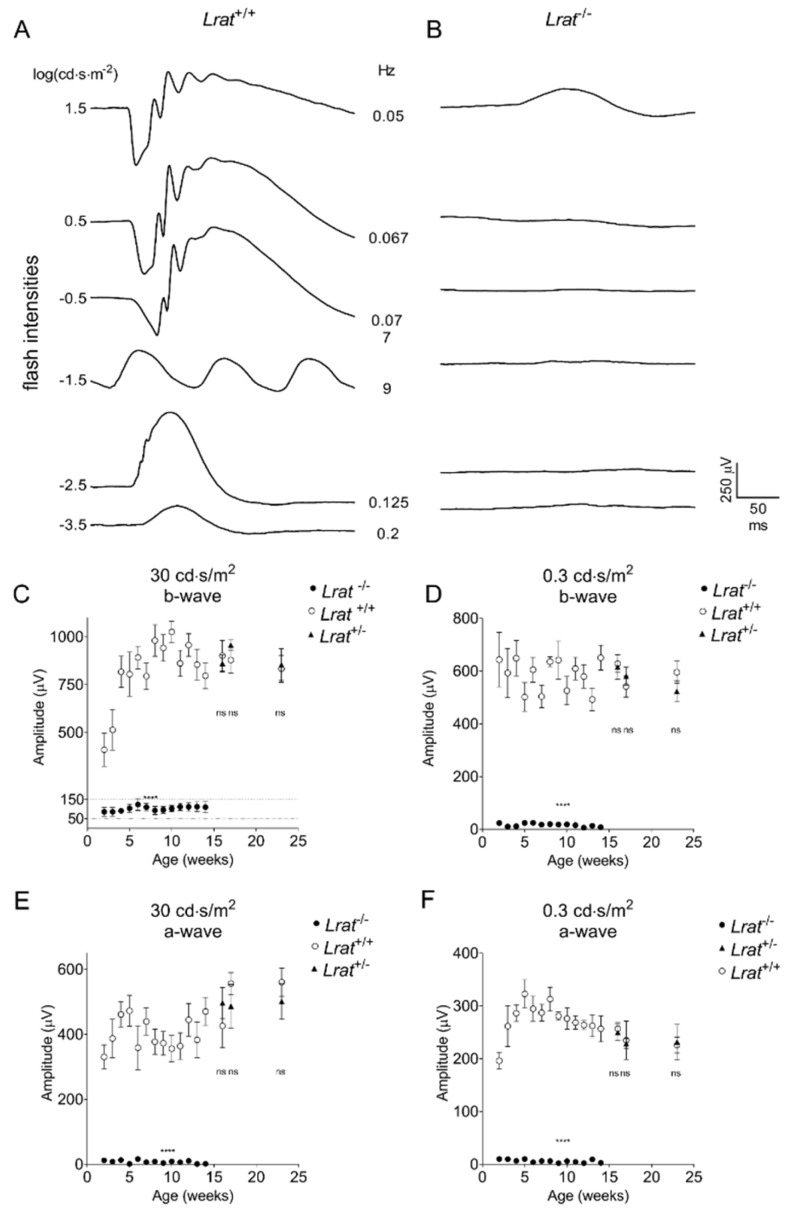
ERG responses of *Lrat*^+/+^ (*n* = 6), *Lrat*^+/−^ (*n* = 5), and *Lrat*^−/−^ (*n* = 6) animals after a single light flash. Averaged traces with increasing flash intensities are plotted for *Lrat*^+/+^ (**A**) and *Lrat*^−/−^ animals (**B**) at 5 weeks of age. A-wave (**C**,**D**) and b-wave (**E**,**F**) amplitudes (µV) are plotted versus the age of the animals after a high light intensity flash (30 cd·s/m^2^) (**C**,**E**) and a lower light intensity flash (0.3 cd·s/m^2^) (**D**,**F**). No significant difference can be observed between the amplitudes of *Lrat*^+/+^ (white circles) and *Lrat*^+/−^ animals (triangles). *Lrat*^−/−^ animals (black circles) have extremely decreased or not detectable ERG responses from 2 weeks of age onwards (**C**–**F**). No dark-adapted a-wave responses were recorded at all, and no b-wave response for 0.3 cd·s/m^2^. ns = non-significant; **** = *p* < 0.0001.

We performed electrophysiological studies to assess the visual function of *Lrat*^−/−^, *Lrat*^+/−^ and *Lrat*^+/+^ rats. We measured and analyzed the responses to scotopic stimuli at regular time points between 2 and 23 weeks of age. ERG traces are presented for *Lrat*^+/+^ and *Lrat*^−/−^ animals at 5 weeks of age (Figure 7A,B), showing several differences that were observed, including the complete absence of recordable a-waves, the almost complete lack of the b-waves, the absence of recordable oscillatory potentials, and the absence of a recordable response to a flicker (9 Hz) in *Lrat*^−/−^ animals.

**Figure 8:**
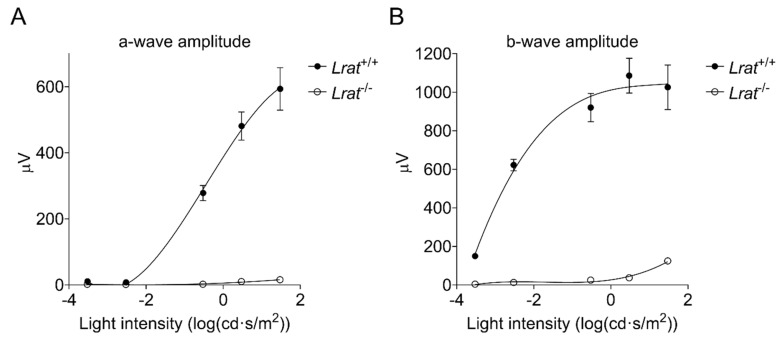
ERG responses of *Lrat*^+/+^ (*n* = 6) (black circles) and *Lrat*^−/−^ (*n* = 6) (white circles) animals at 2 weeks of age after a single light stimulus. Averaged a-wave (**A**) and b-wave (**B**) amplitudes per experimental group plotted against increasing light intensity. B-wave responses were extremely decreased, and a-wave responses were almost completely absent in *Lrat*^−/−^ animals, indicating that these animals are blind from 2 weeks of age onwards.

Wildtype control animals of various ages did not show any significant differences in ERG amplitudes between time points (*n* = 6; see Supplementary Figure S4). In contrast, differences were observed for the *Lrat*^−/−^ animals. Hardly any responses were observed for this group at all time points. Only at the highest light intensity flash (30 cd·s/m^2^) we observed a slight response for *Lrat*^−/−^ animals. Dark-adapted a-wave amplitudes (Figure 7E,F) and b-wave amplitudes (Figure 7C,D) were quantified over time and presented in Figure 7D,F for 0.3 cd·s/m^2^ and in Figure 7C,E for 30 cd·s/m^2^. From the age of 2 weeks onwards, dark-adapted a-wave amplitudes were hardly recordable in *Lrat*^−/−^ animals and b-wave amplitudes were strongly decreased in *Lrat*^−/−^ animals compared to *Lrat*^+/+^ animals. At 5 weeks of age, dark-adapted a-wave amplitudes were hardly recordable, and b-wave amplitudes were strongly decreased for *Lrat*^−/−^ animals for all light intensities (Figure 8A,B). We also performed ERGs in heterozygous (*Lrat*^+/−^) animals. However, no significant differences were observed between *Lrat*^+/−^ and *Lrat*^+/+^ animals. The relevant data are presented in Figure 7C–F and Supplementary Figure S5.

### 2.5. Vision-Based Behavioral Analysis

**Figure 9:**
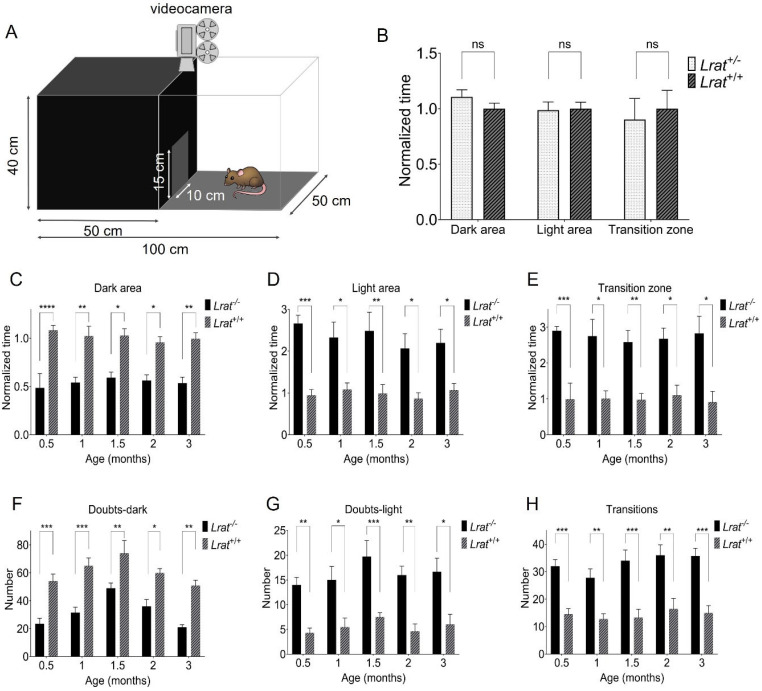
Light/dark box vision-based behavioral responses of *Lrat*^+/+^ (*n* = 6), *Lrat*^+/−^ (*n* = 5) and *Lrat*^−/−^ (*n* = 6) animals. A schematic overview of the customized light/dark box used is presented in panel (**A**). No significant differences between *Lrat*^+/−^ and *Lrat*^+/+^ animals were observed for the time spent in any area at the age of 23 weeks (**B**). *Lrat*^−/−^ spend more time in the dark area than the *Lrat*^+/+^ animals (**C**,**D**). *Lrat*^−/−^ spend more time in the transition zone (the area around the door) and switch compartment more than *Lrat*^+/+^ animals (**E**). *Lrat*^+/+^ animals doubt more to enter the light compartment (**F**) and less to enter the dark compartment (**G**) than *Lrat*^−/−^ animals. *Lrat*^−/−^ switch areas more often than *Lrat*^+/+^ animals (**H**). Wildtype rats are more comfortable spending more time in a dark area. These data show that the knockout animals do not have the same preference, indicating that they cannot distinguish between the dark and light areas and are visually impaired. The data in panels (**B**–**E**) are normalized to the averaged *Lrat*^+/+^ behavior of all measurements for each parameter separately. The wildtype animals’ data were averaged for all measurements (all time points together) per parameter. This was possible since the behavior of the wildtype animals did not change significantly over time. These values were used for the normalization of the data of the *Lrat*^−/−^ animals. ns = non-significant; * = *p* < 0.05; ** = *p* < 0.01; *** = *p* < 0.001; **** = *p* ≤ 0.0001.

We performed a light/dark box vision-based behavioral assay with *Lrat*^+/+^, *Lrat*^+/−^, and *Lrat*^−/−^ animals at different ages (Figure 9). We did not observe significant differences between *Lrat*^+/+^ and *Lrat*^+/−^ for several test parameters such as time spent in the dark area, the light area, and the transition zone (Figure 9B). Clear and significant differences were observed between *Lrat*^+/+^ and *Lrat*^−/−^ animals (Figure 9C–H). *Lrat*^−/−^ animals usually spend less time in the dark area (Figure 9C), more time in the light area (Figure 9D), and more time in the transition zone (Figure 9E) compared to *Lrat*^+/+^ animals. Wildtype animals are more resistant to entering the lighted compartment (Figure 9F) compared to knockouts and less resistant to entering the darkened compartment (Figure 9G). Moreover, *Lrat*^−/−^ animals changed compartments more than *Lrat*^+/+^ animals (Figure 9H). Altogether, these results indicate that *Lrat*^−/−^ animals cannot distinguish between the light and the dark area of the box and, thus, do not prefer where to spend most of their time. Wildtype and heterozygote animals prefer to spend their time in the dark area, as expected from rats with normal vision.

## 3. Discussion

In this study, we generated and phenotyped a new rat model for RP based on a mutation in the *LRAT* gene in our hospital’s RPA patient group [4,13]. The rat equivalent of the human mutation (c.12delC) in *Lrat*, c.12delA, was successfully introduced in the Brown Norway rat strain using CRISPR/Cas9-based gene editing. The expression of *Lrat* was found in several tissues, including the liver, lung, testis, and eye, thereby confirming data from the literature [15,16,17,34]. Homozygous *Lrat* mutants were followed up by SLO-OCT, ERG, and vision-based behavioral tests.

We confirmed that the targeted mutation in both genomic DNA as cDNA in our *Lrat*^−/−^ animals was the causative mutation for the in vivo vision-related phenotype that we observed in this strain. The introduced mutation (*Lrat* c.12delA) causes a frameshift and a predicted premature chain termination at position 72 (p.M5CfsX72). The mRNA is possibly degraded by nonsense-mediated decay [35]. Indeed, we observed significantly less mRNA expression in *Lrat*^−/−^ tissues compared to the wildtype tissues. Immunohistochemistry data on liver sections showed the absence of LRAT protein. The remaining expression of *Lrat* mRNA in our knockout rat is an observation that we cannot explain readily yet and is a subject for further studies. Nonetheless, it is well known that a small subset of specific mRNA species containing a premature chain termination escapes nonsense-mediated decay [35,36,37,38,39]. Another possible explanation might be related to the complex and partly unknown transcriptional machinery of the *Lrat* gene. Zolfaghari and coworkers (2002) found that the regulation of *Lrat* expression in different tissues is complex and occurs through a combination of mechanisms. They identified several potential signals for polyadenylation in the 3’UTR region of the *Lrat* transcript resulting in the expression of alternative smaller transcripts in specific tissues [17]. Possibly, minute quantities of alternatively spliced or regulated *Lrat* mRNA are induced by knocking out the gene, a phenomenon called transcriptional modulation. Last but not least, our mutated *Lrat*^−/−^ rat strain and patients harboring an *LRAT* mutation do not show an apparent additional phenotype besides vision-related problems. *Lrat* deficiency did not appear to adversely affect the long-term survival or fertility of male or female *Lrat*^−/−^ rats. This suggests that the lack of functional LRAT protein might be compensated in other tissues besides the eye. Future in-depth studies on (the regulation of) *Lrat* expression and translation in the retina and other tissues may shed light on these issues.

In this study, we report the disease course of a new rat model for RP in detail using structural and functional phenotypic assessment of the retina and vision. In summary, the single nucleotide deletion in our *Lrat*^−/−^ rat resulted in functionally blind rats, as measured by ERG and vision-based behavior assays, from two weeks of age onward. The functional retinal abnormalities measured by ERG precede the structural abnormalities as measured by OCT. Retinal degeneration started within three weeks of age (OCT) in *Lrat*^−/−^ rats. At four months of age, their retinal thickness was significantly reduced to roughly 80% of the original thickness. Our *Lrat*^−/−^ rat’s phenotype is highly similar to that of a previously published *Lrat*^−/−^ mouse model. This mouse model harbors a targeted mutation in which the whole first exon of the *Lrat* was replaced with a neomycin cassette. Similar to our rat model, the homozygous *Lrat*^−/−^ mice are viable and fertile but blind from early-onset [6]. At the age of 6–8 weeks, in *Lrat*^−/−^ mice, histological analysis revealed shorter (35%) retinal rod outer segments than the wildtype controls. The *Lrat*^−/−^ mice lost sensitivity of pupillary light responses and had abnormal electroretinograms. Besides thinning of the retina, relatively few morphological changes in the *Lrat*^−/−^ mouse retina were visible from OCT images at an early age (6–8 weeks) under normal laboratory circumstances [6,19]. We obtained comparable results using OCT imaging in our *Lrat*^−/−^ rat.

Although the *Lrat* c.12delA rat mutation was designed after a genetic defect in a human patient group, there is a considerable difference in the disease’s onset and progression in humans and mice. The phenotype of patients harboring *LRAT* mutations is variable and relatively mild. Within the cohort carrying the c.12delC mutation, the first symptom usually presents itself within the early years of life, generally starting with nyctalopia. Later, patients have significantly decreased scotopic ERG measurements and an overall decrease in visual sensitivity. OCT showed normal architecture of the retinal layers. Funduscopy revealed significant variability in the quantity of white dots observed in the (mid-)peripheral fundus, which seemed to be dome-shaped hyperreflective lesions extending from the RPE as determined using OCT [4]. The mean age of reaching blindness or severe visual impairment varies between 50 and 60 years of age [13]. In other patient cohorts harboring other pathogenic *LRAT* mutations, this is broader: between childhood to 60 years of age [2,13,40]. In contrast to the clinical picture in our human patient group, both our new *Lrat*^−/−^ rat and the previously published mouse strains reach functional blindness and retinal degeneration much sooner, during infancy if not directly after birth. Mice and rats open their eyes between 14–16 days of age. The phenotypic difference could perhaps be explained by the difference between rodent and human eyes. Since mice and rats are nocturnal mammals and humans are diurnal, mice and rats have many more rods than cones. Rods are more efficient in responding to low light intensity conditions than cones. However, cones allow for greater visual acuity [41,42,43]. *Lrat*^−/−^ rodents reach blindness relatively earlier in life than human patients. However, the first symptom for patients harboring an *LRAT* mutation usually is nyctalopia during infancy within the first year of life [13]. This can be explained by the fact that the rat’s retina contains many more rods, which function better in lower light, than cones, which are responsible for color vision and work best in bright light. The absence of scotopic ERG responses in both rodents and humans lacking LRAT is a common characteristic. Finally, besides the absence of scotopic ERG responses, another similarity is the progressive retinal thinning as judged from OCT scans. In both human patients and our *Lrat*^−/−^ rat strain, the ONL progressively degenerates [13].

Our newly developed model can be used for the development of therapeutic approaches for the *LRAT*-subtype of RP. This subtype is, at present, an incurable disease, as are most forms of RP. The only exception is the *RPE65*-subtype of RP, which is highly related to the *LRAT*-subtype [13]. For the *RPE65*-subtype of RP, AAV2-mediated augmentation gene therapy (voretigene nepravovec) is currently on the market [44,45]. From a biological point of view, the duration of the treatment effect is expected to last a lifetime. However, since extended patient follow-up data of treated patients are still scarce, there are still some uncertainties about the long-term impact of voretigene nepravovec [44]. Talib and colleagues (2019) suggested that patients with retinal degenerations caused by *LRAT* mutations may be particularly susceptible to treatments, such as gene-replacement therapy, since not (all) pathogenic mutations lead to early onset of severe visual dysfunction. Indeed, the patients suffering from RD, caused by the c.12delC mutation in *LRAT*, show long-term preservation of the outer retina, at least at the level of the fovea. This is potentially a favorable finding, as indicated by positive results of an ongoing therapeutic clinical trial (NCT01256697) in which patients with *RPE65*- and *LRAT*-subtype of RP receive oral synthetic 9-cis-retinoids [13]. In contrast to the *RPE65*-subtype of RP, the window of therapeutic opportunity for *LRAT*-RP patients can thus be extended to later decades of life [13]. However, the best therapeutic window in these patient groups needs to be determined still [46].

Since *Lrat* is expressed in the RPE, subretinal injections are more preferential over intravitreal injections for experimental therapies such as gene- and cell-replacement therapy. In mice, subretinal injections are generally challenging to perform with lower success rates than intravitreal injections. Moreover, it was recently suggested that suprachoroidal injections are possibly even more efficient than intravitreal and/or subretinal injections to target the RPE [47]. Suprachoroidal injections are a relatively new concept and need to be developed and fine-tuned in rodents. Experimental cell-based therapeutic studies may require either ocular injections or subretinal surgery. In vivo cell-therapeutic studies targeting (*Rpe65*^−/−^ and *Lrat*^−/−^ mediated) damage of RPE cells using injections of dissociated (RPE) cells resulted in variable outcomes [28,48,49]. More promising results were obtained with RPE cells transplanted as a monolayer with or without a carrier [50,51,52,53,54]. The procedures for transplanting cells as a sheet are more extensive than a relatively simple single subretinal injection. To transplant these tissues and scaffolds into mice’s eyes is a significant, maybe not to overcome, technical challenge. Given the fact that the rat eye’s volume (±50–55 µL) is roughly ten times as large as the mouse eye’s volume (±4–5 µL) [55,56], in our hands, (all) ocular interventions are performed with a much higher success rate in rat eyes. We think that the more complex ocular interventions, such as sheet transplantations, are possible in rats’ eyes, but not mice’s eyes, given the currently commercially available instruments, indicating the need for larger eyed models, such as rats, to facilitate the exploration of novel therapies for RDs. Moreover, rats usually are the leading model for studies of physiology, pharmacology, toxicology, and neuroscience [57]. Indeed, it was suggested that rats are superior to mice as models for humans in neuroscience and behavioral assays [58,59,60]. Taken together, both the eye size and visual evaluation of (genetically manipulated) rats have substantial advantages over commonly used mice in preclinical studies studying RDs.

The number of suitable rat models for RP is scarce. We compared the retinal features of our *Lrat*^−/−^ rat with those of the widely used Royal College of Surgeons (RCS) rat, which was the first described, and is a commonly used animal model for inherited retinal degeneration [30,31,61,62,63,64,65,66,67]. Additionally, it has been extensively used for testing the efficiency of the transplantation of RPE cells [32]. The RCS rat harbors a (spontaneous) mutation in the *Mertk* gene, which is uniquely and highly expressed in the RPE. *Mertk* deficient animals fail to phagocytose shed PR outer segments that accumulate in the subretinal space. Subsequently, the PRs die, which may interfere with the assessment of therapeutic strategies. Despite the apparent advantages of a rat model with larger eyes, photoreceptor debris accumulates in the subretinal space, initiating spontaneous retinal degeneration and hampering experimental treatment modalities [33].

In conclusion, our newly developed *Lrat*^−/−^ rat model is based on an existing patient population. It is an RD model without debris in the subretinal space. Crucially, the rat eye is large enough to perform complex procedures such as subretinal injections, suprachoroidal injections and RPE sheet transplantations. This model will be very useful for developing therapeutic approaches and determining therapeutic windows for this patient group, and possibly also for other RDs.

## 4. Methods

### 4.1. Construction of the Animal Model

All animal experiments were conducted following the ARVO Statement for the Use of Animals in Ophthalmic and Vision Research and approved by the Netherlands’ national committee. The previously detected deletion of a cytosine (C) of the 12th base pair (c.12delC) in the coding region of *LRAT* leads to a frameshift and a premature stop codon in exon 1 (p.M5CfsX53). Homology analysis between the human *LRAT* and rat *Lrat* gene showed that the rat’s equivalent of the human c.12delC mutation is c.12delA. This deletion is predicted to have a similar truncating effect (p.M5CfsX72) on the protein as the human variant. The *Lrat* mutant model (Brown Norway background) was produced in collaboration with GenOway (France) using a CRISPR/Cas9 approach to introduce the mutation according to the protocols published elsewhere [68,69]. The Cas9 nuclease and single guide RNA (sgRNA) were used to edit the *Lrat* gene localized to chromosome 2 (2q34). sgRNA was designed to target exon 1 of the *Lrat* gene using CRISPOR.org (http://crispor.tefor.net/; accessed on 16 February 2017), a web-based tool to select CRISPR/Cas9 target sequence: 5′-AAGGATGAAGAACTCAATGC-3′. Six predicted (27 September 2017) off-target sites have been identified from internal genOway process: *Chr1:212689122-212689137*; *Chr2:150627149-150627164*; *Chr2:181905085-181905100*; *Chr9:60433155-60433170*; *Chr16:64050524-64050539*, *ChrX:28904421-28904436* (*genome* assembly *Rnor* 6.0). A short homologous single-stranded oligonucleotide (ssODN) carrying the c.12delA point mutation (CAGTTGCGGCCAGCGAGAAACTCTGGTCTTTAAAGGATGAAGAACAGTTGCTGGAGGCTGCGTCCCTCCTTCTGGAGAAGCTGCTCCTTATTTC) was used as a template for homology-directed repair. sgRNA and ssODN were ordered from Integrated DNA Technologies (IDT, Coralville, IA, USA). Fertilized oocytes were collected from superovulated Brown Norway female rats previously mated with males. The gRNA (1 µM), ssODN (0.6 µM) and the Cas9 nuclease (0.4 µM, Alt-R^TM^ S.p. Cas9 Nuclease V3, IDT) were then microinjected into the male pronucleus. Injected zygotes were cultivated overnight to the two-cell stage to assess sgRNA toxicity. The resulting two-cell embryos were reimplanted into pseudopregnant foster mothers at 0.5 days post-coitum. Standard surveyor assays were used to detect insertions and/or deletions at the targeted site in the genome. The targeted locus was amplified and sequenced to identify point mutant animals. Two founders harboring the c.12delA point mutation (*Lrat*^−/−^) were identified and bred to generate heterozygous animals (*Lrat*^+/−^). PCR amplification and Sanger sequencing of each predicted off-target site was realized, and no polymorphism was identified in the F1 generation. *Lrat*^+/−^ animals were used to expand a colony at our local animal facility. Homozygous *Lrat* knockout rats (*Lrat*^−/−^) and their heterozygote (*Lrat*^+/−^) and wildtype control (*Lrat*^+/+^) littermates were born and reared at the VU University, Amsterdam. All animals were kept on a light cycle of 12 h on/12 h off and were fed ad libitum. The animals were followed over time for visual examination. Both males and females were used in these studies. At the start of the experiments, the rats were 15 days old, weighing 30–40 g.

### 4.2. Mutation Analysis and Expression of the Lrat Gene

According to standard protocols, we isolated genomic DNA from ear snips between 10 and 14 days of age using phenol extraction [34]. To confirm the introduced mutation in the experimental animals, we used PCR and sequencing. By PCR, a 365 bp product surrounding the point mutation was generated using forward primer (in 5’–3’ direction) GCTGACCAACACTACATCCTC and reverse primer GGGTCCGTGACACTTCCAAC. The fragment was sequenced (BigDye^TM^) and analyzed using CodonCode software according to the manufacturer’s protocol. 

*Lrat*^+/+^, *Lrat*^+/−^, and *Lrat*^−/−^ animals were terminated using CO_2_ gas at 2.5 months of age. The liver, kidney, spleen, small intestine, lung, eye, brain, and testis were collected and placed on dry ice immediately. Total RNA was extracted using TRIzol Reagent (Ambion^TM^) according to the manufacturer’s protocol. The Nano-drop (ND-1000) was used to check the RNA’s concentration and quality, and 200 ng total RNA was used for cDNA synthesis using Superscript III (Invitrogen^TM^) and an oligodT according to the manufacturer’s protocol. *Lrat* RNA presence was checked using two sets of specific primers for rat *Lrat* mRNA (from 5’–3’ set 1: forward GCAGATACGGCTCTCCTAT; and reverse GCCAGACATCATCCACAAGC, and set 2: forward ACCTTGCACAGACCAGTTGC; and reverse: CAGTCTCGTGAAACTTCTC). The product was sequenced using Bigdye^TM^ (according to the manufacturer’s protocol), and the mutated sequence in *Lrat*^−/−^ and *Lrat*^+/−^ animals was confirmed. PCR reactions of the cDNA were performed using a multiplex set-up in which *Ef1a* served as a reference gene. In one PCR reaction, the *Lrat* and *Ef1a* PCR products were generated. The primers for *Ef1a* were made in an exon spanning design (from 5’–3’ forward: CTGGCTTCACTGCTCAGGTG; and reverse: GGCTTGCCAGGGACCATGTC).

### 4.3. LRAT Detection Using Immunofluorescence

*Lrat*^+/+^ and *Lrat*^−/−^ animals were terminated using CO_2_ gas at 2.5 months of age. The liver was collected and placed on dry ice immediately. Tissues were embedded in Optimal Cutting Temperature (O.C.T.)^TM^ Compound (Tissue-Tek^®^) and cut into 5 µm sections using a cryostat ultramicrotome. The sections were fixed in 4% PFA in 1x PBS at room temperature (RT) for 10 min. Incubation with the primary antibody for LRAT (1:200, custom-made by Biomatik, Kitchener, ON, CA) was performed in blocking buffer (1% BSA, 0.2% Triton^TM^ X-100 in 1x PBS) for 90 min at RT. The secondary antibody (1:200, Goat-anti-Rabbit-Cy3, 111-166-003, Jackson ImmunoResearch, Ely, UK) was incubated in 0.2% Triton^TM^ in 1x PBS for 45 min in the dark at RT. The sections were embedded in Vectashield^®^ mounting medium with DAPI (H-1200, Vector Laboratories, Burlingame, CA, USA) and imaged using a Leica TCS SP8 X confocal microscope.

### 4.4. The Experimental Set-Up, Randomization, Blinding, and Drop-Outs

Animals were given an identification number before entering the experiment. Investigators and care-takers were blinded for their genotype (e.g., experimental group). Males and females were housed separately in groups. Cage arrangements were determined randomly using the randomizing function of Microsoft Excel. Animals were followed over time using scanning laser ophthalmoscopy (SLO), optical coherence tomography (OCT), electroretinography (ERG), and vision-based behavioral measurements. At the start of every measurement, the measuring order was randomly determined using the randomizing function of Microsoft Excel. The rats were measured using SLO-OCT and ERG at a wide range of ages. *Lrat*^−/−^ animals (*n* = 6) were measured weekly from 2 weeks of age onwards. *Lrat*^+/−^ (*n* = 5) animals were measured at 16, 17, and 23 weeks of age only. Since only patients with a homozygous, and not heterozygous, c.12delC mutation suffer from RD, we did not expect a difference in these animals’ visual phenotype compared to the *Lrat*^+/+^ animals (*n* = 6) beforehand. A light/dark-box assay was done at 0.5, 1, 1.5, 2, and 3 months of age. At the end of the study, all animals were sacrificed, and the eyes and other tissues were collected for additional in vitro analyses. There were no drop-outs throughout the experiment.

### 4.5. Scanning Laser Ophthalmoscopy (SLO) and Optical Coherence Tomography (OCT) Measurements

SLO uses laser scanning microscopy to obtain images (fundus photos) of the retinal surface. OCT uses near-infrared light to obtain high-resolution two- and three-dimensional images within the retina. SLO-OCT measurements were performed using a commercially available system (Heidelberg Engineering Spectralis combined HRA+OCT, Heidelberg, DE), modified for use with animals (Medical Workshop, Utrecht, NL). Detailed methods are described elsewhere [35,36]. In short, animals were anesthetized with an intraperitoneal injection of a mixture of ketamine (22 mg/kg for animals <4 weeks of age; 65 mg/kg for animals ≥4 weeks of age) and xylazine (2.2 mg/kg for animals <4 weeks of age; 7.5 mg/kg for animals ≥4 weeks of age) diluted in 0.9% NaCl. The eyes were locally anesthetized using tetracaine-hydrochloride drops (1% *w*/*v*) and were dilated using tropicamide (0.5% *w*/*v*), and atropine (1% *w*/*v*) drops. Hylocomod drops were applied to maintain corneal hydration at all times. A contact lens (5.2 mm in diameter; Cantor-Nissel, Brackley, UK) was placed. The standard 30° field of view equipment set was used. Animals were placed on a custom-made heated holder, eyes were kept moist using Hylocomod eye drops, and body temperature was monitored. Imaging was done using the Eye Explorer software version 1.9.14.0 (Heidelberg Engineering, Heidelberg, DE). For fundus images, infra-red (820 nm) intensity was manually adjusted to prevent overexposure. OCT imaging was performed using a volume scan (57 frames, 786 A-scans, 30° × 25°, 61 scans, Δ120 µm, 8.8 scans per second). The reference arm was manually adjusted according to the manufacturer’s instructions. The follow-up function was used whenever possible to ensure accurate thickness profiles between time points. Frame analysis was done on a total of five single OCT scans and corresponding thickness profiles. The chosen scans were the crossing of the optic nerve (ON = 0), the middle superior section (ON + 10), the superior section (ON + 20), the middle inferior section (ON - 10), and the inferior section (ON - 20). Within each selected OCT scan, retina thickness was determined on 1 mm intervals, with a 0.5 mm minimum distance from the optic nerve. The thickness values were averaged, normalized, and shown ± the standard error.

### 4.6. Electroretinography (ERG)

Using ERG, the electrical activity of the retina in response to a light stimulus is measured. The ERG arises from currents generated by the retinal neurons and glia. The animals were kept in total darkness in their home cage for at least 1 hour before scotopic ERG measurements and were anesthetized with an intraperitoneal injection of a mixture of ketamine (22 mg/kg for animals <4 weeks of age; 65 mg/kg for animals ≥4 weeks of age) and xylazine (2.2 mg/kg for animals <4 weeks of age; 7.5 mg/kg for animals ≥4 weeks of age) diluted in 0.9% NaCl. The eyes were locally anesthetized using tetracaine-hydrochloride drops (1% *w*/*v*) and were dilated using tropicamide (0.5% *w*/*v*), and atropine (1% *w*/*v*) drops. Hylocomod drops were applied to maintain corneal hydration at all times. The animals were placed in the RETImap full flash Ganzfeld (Roland Consult, Brandenburg an der Havel, DE) using a carrier table, which was kept at 37 °C. Body temperature was carefully monitored during all measurements. ERGs were recorded using gold electrodes, which were placed on the corneas of both eyes. Another gold electrode was placed in the animal’s mouth serving as a reference for both eyes simultaneously. A needle was placed subcutaneously near the tail, which served as a ground electrode. See Supplementary Table S1 for the light intensities, the number of flashes used for averaging, and the flashes’ interval. ERG traces were 350 ms long, utilizing 512 data points.

All ERG data were systematically analyzed, without human intervention, using a custom-made Matlab script. The data was zero-centered by averaging the signal before the stimulus (<20 ms) and subtracting the resultant from the entire trace. A low-pass filter (4th order, 30 Hz (for the b-wave) and 235 Hz (for the a-wave)) was applied in both the forward and backward direction to remove noise and the oscillatory potentials (OPs). 30 Hz is well below the minimum expected frequency, and 235 Hz resembles the expected maximum frequency of OPs in rats [37]. The *findpeaks* function in Matlab was used to find the latencies of the a- and b-waves in the filtered data. The magnitudes of the unfiltered signal at the selected latencies were characterized as the values for b-wave and the absolute a-wave amplitude. The absolute a-wave was subtracted from the value of the b-wave amplitude to calculate the absolute b-wave amplitude. Flicker properties were determined from the original, unfiltered trace. The time to the first peak (P1) and the second peak amplitude (P2) were identified. The (absolute) b-wave, a-wave, and flicker properties of each group, at each age, were averaged and normalized to the corresponding 30 cd· s/m^2^ response from the wildtype control group (Supplementary Figure S5).

### 4.7. Light/Dark-Box Behavioral Assay

At the ages of 0.5, 1, 1.5, 2, and 3 months (for *Lrat*^−/−^ and *Lrat*^+/+^ animals) and 5 months (for *Lrat* ^+/−^ and *Lrat*^+/+^), a vision-based behavioral assay was performed. A customized light/dark-box was used with dimensions of 100 × 50 × 40 cm (length × width × height). Half of the box was darkened. The box was placed in the same position in the room during every measurement to prevent possible light/shade interference. The animals were placed in the light area of the box and filmed for twenty minutes. Deep learning was used to extract key features. In short, a Faster Recursive Convolutional Neural Networks (Faster R-CNN) was used to locate and track the rat’s head. The Faster R-CNN was developed using the resnet18 architecture and trained on 658 randomly sampled, annotated video frames. After the detector was trained, it was deployed on each video. A transition zone, dark zone, and light zone were determined. The transition zone was defined as a circle centered at the doorway base with a radius of 1.25 times the doorway’s width. The rat was tracked in the light/dark box, and per frame, the rat’s location was tracked. After tracking the rat’s heads in each video, the data were processed using Matlab. A random subset of videos was selected to manually extract all parameters and compare the data to the values extracted via the Faster R-CNN-based algorithm. No significant differences were found between the manually extracted and automatically extracted parameters, confirming the automatic analysis’s robustness.

### 4.8. Statistical Analyses Performed

Data were analyzed using one- or two-way ANOVA analyses, ANOVA analyses of the log-transformed data, and the Kruskal-Wallis analyses with posthoc Bonferroni to determine the statistical significance of all data. Similar *p*-values were obtained using all tests. *p*-values are reported: *: *p* ≤ 0.05, **: *p* ≤ 0.01, ***: *p* ≤ 0.001, and ****: *p* ≤ 0.0001.

## Data Availability

The data presented in this study are available on request from the corresponding author.

## Supplementary Materials

The following are available online at https://www.mdpi.com/article/10.3390/ijms22137234/s1, Supplementary Table S1: An overview of the scotopic ERG settings which were used throughout the experiment. Supplementary Table S2: The progeny from *Lrat*^+/−^ × *Lrat*^+/−^ breeding results compared to the standard Mendelian frequencies. No significant deviation were observed (*p* > 0.9, according to the Chi-Square Test). Supplementary Figure S1: The weight progression of *Lrat*^−/−^ versus *Lrat*^+/+^ animals (*n* = 3 males and *n* = 3 females) (A) and *Lrat*^+/−^ versus *Lrat*^+/+^ animals (*n* = 3 males and *n* = 2 females) (B). No significant differences in weight progression was observed between the genotypes Supplementary Figure S2: Expression analysis of *Lrat* mRNA in the liver, eyes, testis, brain, kidney, spleen, lung and small intestine of *Lrat*^+/+^ (WT), *Lrat*^+/−^ (HZ) and *Lrat*^−/−^ (KO) rats. *Ef1a* was used as a reference gene. *Lrat* mRNA was abundantly present in the liver, eye and testis. Little expression was seen in lung tissue (yellow arrows), and no expression was observed in the brain, kidney, spleen and small intestine. Less expression of *Lrat* was seen in KO tissues compared to WT and HZ tissues. Supplementary Figure S3: Representative SLO and OCT images of *Lrat*^+/+^ and *Lrat*^+/−^ rats at different ages. It is possible to clearly identify all retinal layers in both the wildtype and heterozygous animals from the OCT images. No differences were observed between the thickness of the retina between wildtype and heterozygous animals. For the quantification, see Figure 6. No significant differences in weight progression was observed between the genotypes Supplementary Figure S4: Single flash ERG responses of increasing light intensities (A and B) of *Lrat*^+/+^ animals at different ages (*n* = 6). The measured a- and b-wave amplitudes at the highest light intensity (30 cd·s/m^2^) is plotted against age (C). No significant differences between the several ages of wildtype animals for a-wave or b-wave amplitudes were observed. Supplementary Figure S5: Scotopic ERG responses of single flash and flicker stimuli. Averaged traces (duration of 350 ms) (*n* = 5 per group) with increasing flash intensities (−3.5–1.5 log(cd·s·m^−2^) are plotted for *Lrat*^+/+^ (A) and *Lrat*^+/−^ animals (B) at the age of 23 weeks. Based on this data, no differences were observed between *Lrat*^+/+^ and *Lrat*^+/−^ animals. Supplementary File 1: The alignment of human and rat wildtype and knockout sequences.

## Abbreviations

LRAT	Lecithin:retinol acetyltransferase
AAV	Adeno-associated virus
ANOVA	Analysis of variance
BSA	Bovine Serum Albumin
c.12delA	Deletion of coding nucleotide 12 adenosine
c.12delC	Deletion of coding nucleotide 12 cytosine
cDNA	Complementary DNA
CRISPR/Cas9	Clustered Regularly Interspaced Short Palindromic Repeats/CRISPR associated protein 9
DAPI	4’,6-Diamidino-2-Phenylindole
DNA	Deoxyribonucleic acid
EF1A	Elongation factor 1-alpha
ERG	Electroretinography
Faster R-CNN	Faster recursive convolutional neural networks
gRNA	Guide RNA
INL	Inner nuclear layer
IPL	Inner plexiform layer
KO	Knockout
LCA	Leber Congenital Amaurosis
Mertk	Proto-oncogene tyrosine-protein kinase MER
mRNA	messenger RNA
O.C.T.	Optimal cutting temperature
OCT	Optical coherence tomography
ONL	Outer nuclear layer
OP	Oscillatory potentials
ORF	Open reading frame
p.M5CfsX53	Frameshift from aminoacid 5 onwards (methionine) resulting in a premature stopcodon 53 aminoacids downstream
p.M5CfsX72	Frameshift from aminoacid 5 onwards (methionine) resulting in a premature stopcodon 72 aminoacids downstream
PBS	Phosphate-buffered saline
PCR	Polymerase chain reaction
PFA	Paraformaldehyde
PR	Photoreceptor
QLT091001	9-cis-retinyl acetate
RBP3	Retinoid-binding protein
RCS	Royal College of Surgeons
RD	Retinal degeneration
RDH	Retinol dehydrogenase
RNA	Ribonucleic acid
RP	Retinitis Pigmentosa
RPA	Retinitis Punctata Albescens
RPE	Retinal pigment epithelium
RPE65	Retinal pigment epithelium-specific protein 65 kDa
RT	Room temperature
RT-PCR	Reverse transcriptase—polymerase chain reaction
SD-OCT	Spectral domain optical coherence tomography
Ser	Serine
sgRNA	Single guide RNA
SLO	Scanning laser ophthalmoscopy
ssODN	Single-stranded oligonucleotide
UTR	Untranslated region
