# Supplementary material for: The Lrat−/− Rat: CRISPR/Cas9 Construction and Phenotyping of a New Animal Model for Retinitis Pigmentosa
Source: Int J Mol Sci. 2021 Jul 5;22(13):7234. doi: 10.3390/ijms22137234 (PMC8267968; doi:10.3390/ijms22137234)
Supplement: Supplementary file 1 [file ijms-22-07234-s001.zip › Supplementary Figures_ Appendix A.pdf]

International Journal of *Molecular Sciences*

MDPI

Supplementary Figures and Tables

Supplementary Table S1: An overview of the scotopic ERG settings which were used throughout the experiment.

Intensity (cd/m²)	Averaged	Interval (Hz)
0.0003	20	0.2
0.003	18	0.125
0.03	14	9 (Flicker)
0.3	15	0.077
3	12	0.067
30	8	0.05

Supplementary Table S2: The progeny from *Lrat*$^{+/-}$ x *Lrat*$^{+/-}$ breeding results compared to the standard Mendelian frequencies. No significant deviation were observed ($p > 0.9$, according to the Chi-Square Test).

Genotype	WT (*Lrat*$^{+/+}$)	Heterozygous (*Lrat*$^{+/-}$)	Homozygous (*Lrat*$^{-/-}$)	Males:Females
Total	34	66	37	64:71
Expected	25%	50%	25%	1:1
Actual	24.8%	48.2%	27.0%	1:1.1

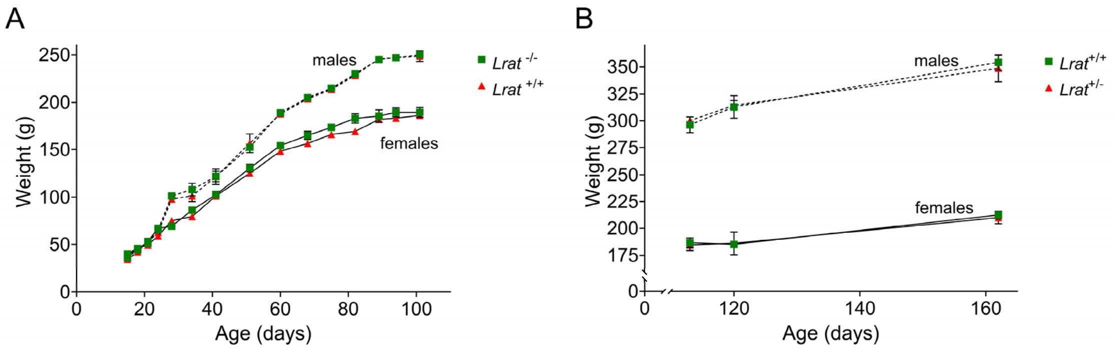

Supplementary Figure S1: The weight progression of *Lrat*$^{-/-}$ versus *Lrat*$^{+/+}$ animals (n = 3 males and n = 3 females) (A) and *Lrat*$^{+/-}$ versus *Lrat*$^{+/+}$ animals (n = 3 males and n = 2 females) (B). No significant differences in weight progression was observed between the genotypes.

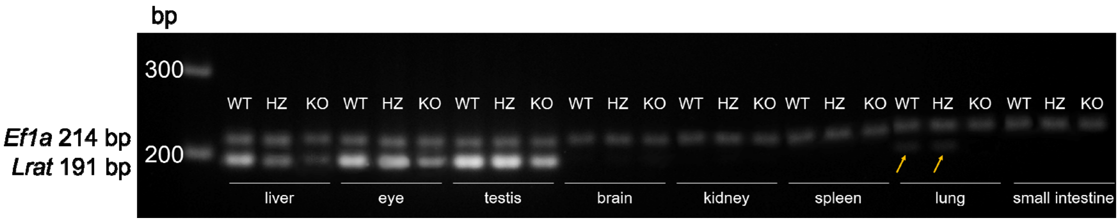

Supplementary Figure S2: Expression analysis of *Lrat* mRNA in the liver, eyes, testis, brain, kidney, spleen, lung and small intestine of $Lrat^{+/+}$ (WT), $Lrat^{+/-}$ (HZ) and $Lrat^{-/-}$ (KO) rats. *Ef1a* was used as a reference gene. *Lrat* mRNA was abundantly present in the liver, eye and testis. Little expression was seen in lung tissue (yellow arrows), and no expression was observed in the brain, kidney, spleen and small intestine. Less expression of *Lrat* was seen in KO tissues compared to WT and HZ tissues.

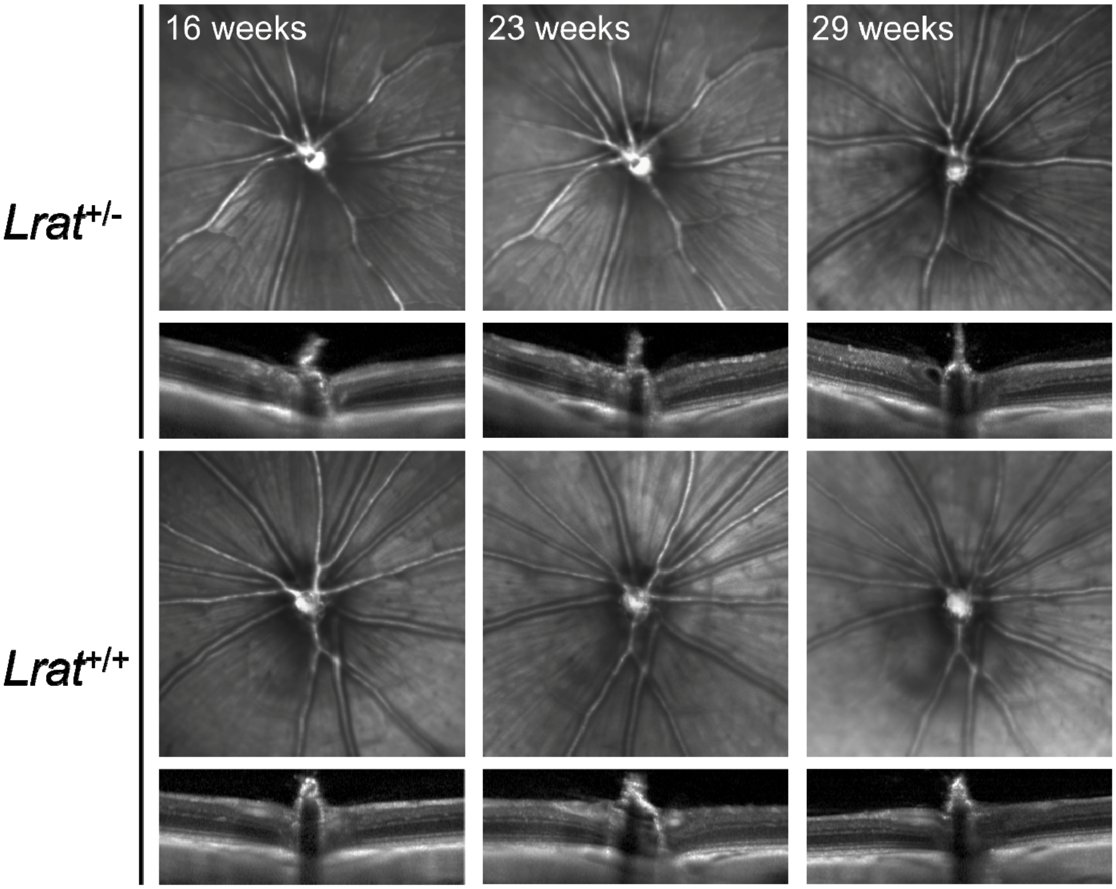

Supplementary Figure S3: Representative SLO and OCT images of $Lrat^{+/+}$ and $Lrat^{+/-}$ rats at different ages. It is possible to clearly identify all retinal layers in both the wildtype and heterozygous animals from the OCT images. No differences were observed between the thickness of the retina between wildtype and heterozygous animals. For the quantification, see **Error! Reference source not found.**. No differences were observed for SLO images as well.

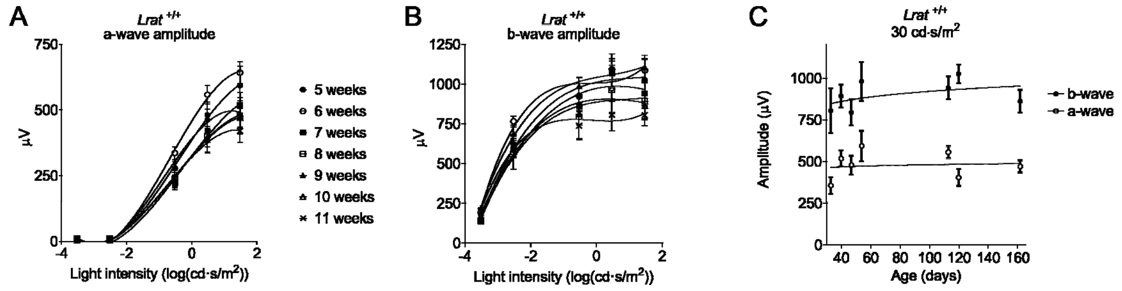

Supplementary Figure S4: Single flash ERG responses of increasing light intensities (A and B) of *Lrat*$^{+/+}$ animals at different ages (n = 6). The measured a- and b-wave amplitudes at the highest light intensity (30 cd·s/m^2) is plotted against age (C). No significant differences (2-way ANOVA) between the several ages of wildtype animals for a-wave or b-wave amplitudes were observed.

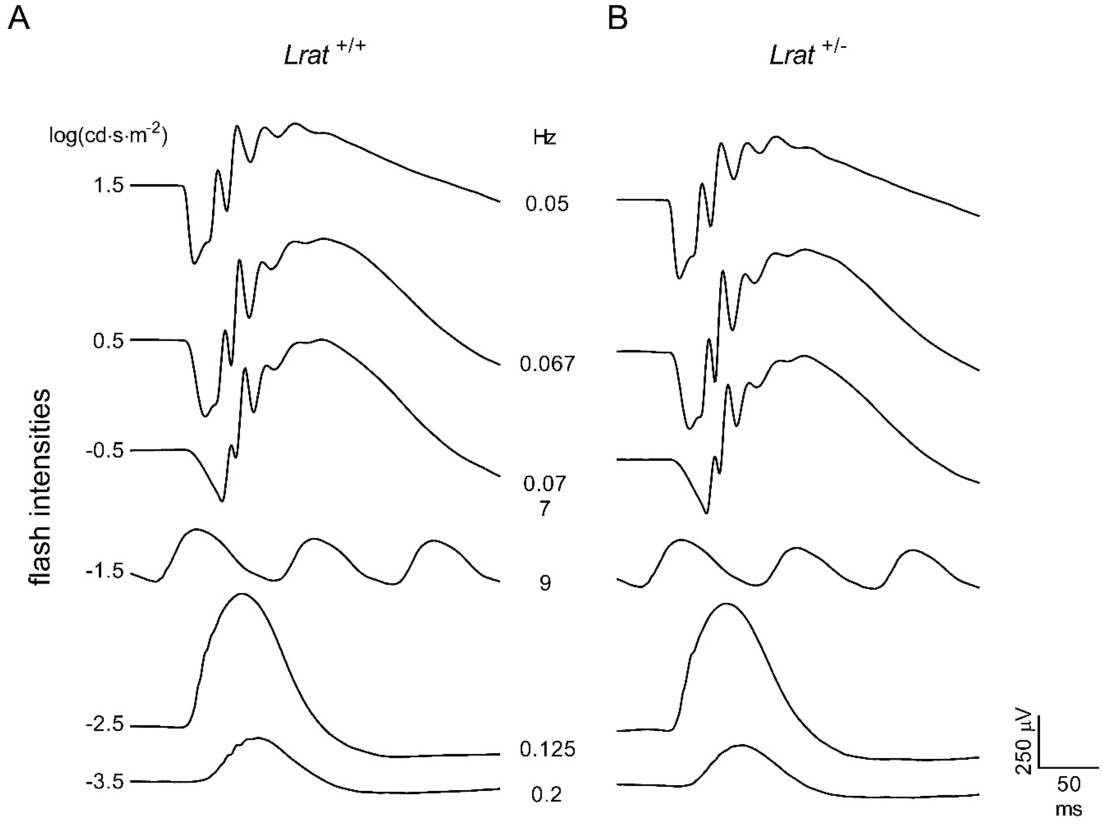

Supplementary Figure S1: Scotopic ERG responses of single flash and flicker stimuli. Averaged traces (duration of 350 ms) (n = 5 per group) with increasing flash intensities (-3.5 – 1.5 log(cd·s·m^{-2}) are plotted for *Lrat*$^{+/+}$ (A) and *Lrat*$^{+/-}$ animals (B) at the age of 23 weeks. Based on this data, no differences were observed between *Lrat*$^{+/+}$ and *Lrat*$^{+/-}$ animals.
