# Supplementary material for: The Lrat−/− Rat: CRISPR/Cas9 Construction and Phenotyping of a New Animal Model for Retinitis Pigmentosa
Source: Int J Mol Sci. 2021 Jul 5;22(13):7234. doi: 10.3390/ijms22137234 (PMC8267968; doi:10.3390/ijms22137234)
Supplement: Supplementary file 1 [file ijms-22-07234-s001.zip › Supplementary file 1_alignments combined.pdf]

Alignment of wildtype and knockout cDNA sequences

```
NM_022290.2    = NCBI reference sequence
ORF            = Open reading frame
###            = Serine

NM_022290.2              CCGCCAGCGAGAAACTCTGGTCTTTAAAGGATGAAGAACTCAATGCTGGAGGCTGCGTCC   300
ORF                      ------------------------------ATGAAGAACTCAATGCTGGAGGCTGCGTCC   30
Wildtype_cDNA            ------------------------------ATGAAGAACTCAATGCTGGAGGCTGCGTCC   30
Knockout_cDNA            ------------------------------ATGAAGAACAGT-TGCTGGAGGCTGCGTCC   29
                                                       *********### *****************

NM_022290.2              CTCCTTCTGGAGAAGCTGCTCCTTATTTCCAACTTCAAGATCTTTAGCGTGTGCGCCCCG   360
ORF                      CTCCTTCTGGAGAAGCTGCTCCTTATTTCCAACTTCAAGATCTTTAGCGTGTGCGCCCCG   90
Wildtype_cDNA            CTCCTTCTGGAGAAGCTGCTCCTTATTTCCAACTTCAAGATCTTTAGCGTGTGCGCCCCG   90
Knockout_cDNA            CTCCTTCTGGAGAAGCTGCTCCTTATTTCCAACTTCAAGATCTTTAGCGTGTGCGCCCCG   89
                         ************************************************************

NM_022290.2              GGAGGAGGCACAGGGAAGAAACATCCCTATGAAATCAACTCTTTTCTCCGGGGTGACGTG   420
ORF                      GGAGGAGGCACAGGGAAGAAACATCCCTATGAAATCAACTCTTTTCTCCGGGGTGACGTG   150
Wildtype_cDNA            GGAGGAGGCACAGGGAAGAAACATCCCTATGAAATCAACTCTTTTCTCCGGGGTGACGTG   150
Knockout_cDNA            GGAGGAGGCACAGGGAAGAAACATCCCTATGAAATCAACTCTTTTCTCCGGGGTGACGTG   149
                         ************************************************************

NM_022290.2              TTGGAAGTGTCACGGACCCATTTTACCCACTATGGGATCTACCTGGGGGACAACCGTGTC   480
ORF                      TTGGAAGTGTCACGGACCCATTTTACCCACTATGGGATCTACCTGGGGGACAACCGTGTC   210
Wildtype_cDNA            TTGGAAGTGTCACGGACCCATTTTACCCACTATGGGATCTACCTGGGGGACAACCGTGTC   210
Knockout_cDNA            TTGGAAGTGTCACGGACCCATTTTACCCACTATGGGATCTACCTGGGGGACAACCGTGTC   209
                         ************************************************************

NM_022290.2              GCCCATCTAATGCCTGACATCCTGTTGGCCCTGACCAGTGACAAGGAACGCACTCAGAAG   540
ORF                      GCCCATCTAATGCCTGACATCCTGTTGGCCCTGACCAGTGACAAGGAACGCACTCAGAAG   270
Wildtype_cDNA            GCCCATCTAATGCCTGACATCCTGTTGGCCCTGACCAGTGACAAGGAACGCACTCAGAAG   270
Knockout_cDNA            GCCCATCTAATGCCTGACATCCTGTTGGCCCTGACCAGTGACAAGGAACGCACTCAGAAG   269
                         ************************************************************

NM_022290.2              GTGGTCTCCAACAAGCGTCTCCTCCCAGGAGTCATTTGCAAGGTGGCCAGCATCCGTGTG   600
ORF                      GTGGTCTCCAACAAGCGTCTCCTCCCAGGAGTCATTTGCAAGGTGGCCAGCATCCGTGTG   330
Wildtype_cDNA            GTGGTCTCCAACAAGCGTCTCCTCCCAGGAGTCATTTGCAAGGTGGCCAGCATCCGTGTG   330
Knockout_cDNA            GTGGTCTCCAACAAGCGTCTCCTCCCAGGAGTCATTTGCAAGGTGGCCAGCATCCGTGTG   329
                         ************************************************************

NM_022290.2              GACACAGTAGAGGACTTTGCCTATGGAGCGGACATCCTCGTCAATCACCTAGACGAGACT   660
ORF                      GACACAGTAGAGGACTTTGCCTATGGAGCGGACATCCTCGTCAATCACCTAGACGAGACT   390
Wildtype_cDNA            GACACAGTAGAGGACTTTGCCTATGGAGCGGACATCCTCGTCAATCACCTAGACGAGACT   390
Knockout_cDNA            GACACAGTAGAGGACTTTGCCTATGGAGCGGACATCCTCGTCAATCACCTAGACGAGACT   389
                         ************************************************************

NM_022290.2              CTCAAGAAGAAGTCCTTGCTCAATGAGGAGGTGGCACGCAGAGCAGAGCAGCAGTTGGGG   720
ORF                      CTCAAGAAGAAGTCCTTGCTCAATGAGGAGGTGGCACGCAGAGCAGAGCAGCAGTTGGGG   450
Wildtype_cDNA            CTCAAGAAGAAGTCCTTGCTCAATGAGGAGGTGGCACGCAGAGCAGAGCAGCAGTTGGGG   450
Knockout_cDNA            CTCAAGAAGAAGTCCTTGCTCAATGAGGAGGTGGCACGCAGAGCAGAGCAGCAGTTGGGG   449
                         ************************************************************

NM_022290.2              CTGACCCCCTACAGCCTACTGTGGAACAACTGCGAACACTTTGTGACCTACTGCAGATAC   780
ORF                      CTGACCCCCTACAGCCTACTGTGGAACAACTGCGAACACTTTGTGACCTACTGCAGATAC   510
Wildtype_cDNA            CTGACCCCCTACAGCCTACTGTGGAACAACTGCGAACACTTTGTGACCTACTGCAGATAC   510
Knockout_cDNA            CTGACCCCCTACAGCCTACTGTGGAACAACTGCGAACACTTTGTGACCTACTGCAGATAC   509
                         ************************************************************

NM_022290.2              GGCTCTCCTATCAGTCCGCAGGCTGAGAAGTTTCACGAGACTGTGAAGATACTCATTCGT   840
ORF                      GGCTCTCCTATCAGTCCGCAGGCTGAGAAGTTTCACGAGACTGTGAAGATACTCATTCGT   570
Wildtype_cDNA            GGCTCTCCTATCAGTCCGCAGGCTGAGAAGTTTCACGAGACTGATAGAAGGAGAGCCGTA   570
Knockout_cDNA            GGCTCTCCTATCAGTCCGCAGGCTGAGAAGTTTCACGAGACTG-----------------   552
                         *******************************************

NM_022290.2              GATCAGAGAAGTTGTCTTGCTTCAGCTGTCTTGGGATTAGTGTCTATTATCTACACAGGC   900
ORF                      GATCAGAGAAGTTGTCTTGCTTCAGCTGTCTTGGGATTAGTGTCTATTATCTACACAGGC   630
Wildtype_cDNA            TGCTGTGGGTGTCA----------------------------------------------   584
Knockout_cDNA            ------------------------------------------------------------   552
```

Alignment of wildtype cDNA sequences using forward and reverse primers

NM_022290.2 = NCBI reference sequence
ORF = Open reading frame

```
ORF                        ------------------------------ATGAAGAACTCAATGCTGGAGGCTGCGTCC   30
NM_022290.2                CCGCCAGCGAGAAACTCTGGTCTTTAAAGGATGAAGAACTCAATGCTGGAGGCTGCGTCC   300
Wildtype_cDNA_forward      ------------------------------ATGAAGAACTCAATGCTGGAGGCTGCGTCC   30
Wildtype_cDNA_reverse      ------------------------------ATGAAGAACTCAATGCTGGAGGCTGCGTCC   30
                                                         ******************************

ORF                        CTCCTTCTGGAGAAGCTGCTCCTTATTTCCAACTTCAAGATCTTTAGCGTGTGCGCCCCG   90
NM_022290.2                CTCCTTCTGGAGAAGCTGCTCCTTATTTCCAACTTCAAGATCTTTAGCGTGTGCGCCCCG   360
Wildtype_cDNA_forward      CTCCTTCTGGAGAAGCTGCTCCTTATTTCCAACTTCAAGATCTTTAGCGTGTGCGCCCCG   90
Wildtype_cDNA_reverse      CTCCTTCTGGAGAAGCTGCTCCTTATTTCCAACTTCAAGATCTTTAGCGTGTGCGCCCCG   90
                           ************************************************************

ORF                        GGAGGAGGCACAGGGAAGAAACATCCCTATGAAATCAACTCTTTTCTCCGGGGTGACGTG   150
NM_022290.2                GGAGGAGGCACAGGGAAGAAACATCCCTATGAAATCAACTCTTTTCTCCGGGGTGACGTG   420
Wildtype_cDNA_forward      GGAGGAGGCACAGGGAAGAAACATCCCTATGAAATCAACTCTTTTCTCCGGGGTGACGTG   150
Wildtype_cDNA_reverse      GGAGGAGGCACAGGGAAGAAACATCCCTATGAAATCAACTCTTTTCTCCGGGGTGACGTG   150
                           ************************************************************

ORF                        TTGGAAGTGTCACGGACCCATTTTACCCACTATGGGATCTACCTGGGGGACAACCGTGTC   210
NM_022290.2                TTGGAAGTGTCACGGACCCATTTTACCCACTATGGGATCTACCTGGGGGACAACCGTGTC   480
Wildtype_cDNA_forward      TTGGAAGTGTCACGGACCCATTTTACCCACTATGGGATCTACCTGGGGGACAACCGTGTC   210
Wildtype_cDNA_reverse      TTGGAAGTGTCACGGACCCATTTTACCCACTATGGGATCTACCTGGGGGACAACCGTGTC   210
                           ************************************************************

ORF                        GCCCATCTAATGCCTGACATCCTGTTGGCCCTGACCAGTGACAAGGAACGCACTCAGAAG   270
NM_022290.2                GCCCATCTAATGCCTGACATCCTGTTGGCCCTGACCAGTGACAAGGAACGCACTCAGAAG   540
Wildtype_cDNA_forward      GCCCATCTAATGCCTGACATCCTGTTGGCCCTGACCAGTGACAAGGAACGCACTCAGAAG   270
Wildtype_cDNA_reverse      GCCCATCTAATGCCTGACATCCTGTTGGCCCTGACCAGTGACAAGGAACGCACTCAGAAG   270
                           ************************************************************

ORF                        GTGGTCTCCAACAAGCGTCTCCTCCCAGGAGTCATTTGCAAGGTGGCCAGCATCCGTGTG   330
NM_022290.2                GTGGTCTCCAACAAGCGTCTCCTCCCAGGAGTCATTTGCAAGGTGGCCAGCATCCGTGTG   600
Wildtype_cDNA_forward      GTGGTCTCCAACAAGCGTCTCCTCCCAGGAGTCATTTGCAAGGTGGCCAGCATCCGTGTG   330
Wildtype_cDNA_reverse      GTGGTCTCCAACAAGCGTCTCCTCCCAGGAGTCATTTGCAAGGTGGCCAGCATCCGTGTG   330
                           ************************************************************

ORF                        GACACAGTAGAGGACTTTGCCTATGGAGCGGACATCCTCGTCAATCACCTAGACGAGACT   390
NM_022290.2                GACACAGTAGAGGACTTTGCCTATGGAGCGGACATCCTCGTCAATCACCTAGACGAGACT   660
Wildtype_cDNA_forward      GACACAGTAGAGGACTTTGCCTATGGAGCGGACATCCTCGTCAATCACCTAGACGAGACT   390
Wildtype_cDNA_reverse      GACACAGTAGAGGACTTTGCCTATGGAGCGGACATCCTCGTCAATCACCTAGACGAGACT   390
                           ************************************************************

ORF                        CTCAAGAAGAAGTCCTTGCTCAATGAGGAGGTGGCACGCAGAGCAGAGCAGCAGTTGGGG   450
NM_022290.2                CTCAAGAAGAAGTCCTTGCTCAATGAGGAGGTGGCACGCAGAGCAGAGCAGCAGTTGGGG   720
Wildtype_cDNA_forward      CTCAAGAAGAAGTCCTTGCTCAATGAGGAGGTGGCACGCAGAGCAGAGCAGCAGTTGGGG   450
Wildtype_cDNA_reverse      CTCAAGAAGAAGTCCTTGCTCAATGAGGAGGTGGCACGCAGAGCAGAGCAGCAGTTGGGG   450
                           ************************************************************

ORF                        CTGACCCCCTACAGCCTACTGTGGAACAACTGCGAACACTTTGTGACCTACTGCAGATAC   510
NM_022290.2                CTGACCCCCTACAGCCTACTGTGGAACAACTGCGAACACTTTGTGACCTACTGCAGATAC   780
Wildtype_cDNA_forward      CTGACCCCCTACAGCCTACTGTGGAACAACTGCGAACACTTTGTGACCTACTGCAGATAC   510
Wildtype_cDNA_reverse      CTGACCCCCTACAGCCTACTGTGGA-----------------------------------   475
                           *************************

ORF                        GGCTCTCCTATCAGTCCGCAGGCTGAGAAGTTTCACGAGACTGTGAAGATACTCATTCGT   570
NM_022290.2                GGCTCTCCTATCAGTCCGCAGGCTGAGAAGTTTCACGAGACTGTGAAGATACTCATTCGT   840
Wildtype_cDNA_forward      GGCTCTCCTATCAGTCCGCAGGCTGAGAAGTTTCACGAGACTGATAGAAGGAGAGCCGTA   570
Wildtype_cDNA_reverse      ------------------------------------------------------------   475

ORF                        GATCAGAGAAGTTGTCTTGCTTCAGCTGTCTTGGGATTAGTGTCTATTATCTACACAGGC   630
NM_022290.2                GATCAGAGAAGTTGTCTTGCTTCAGCTGTCTTGGGATTAGTGTCTATTATCTACACAGGC   900
Wildtype_cDNA_forward      TGCTGTGGGTGTCA----------------------------------------------   584
Wildtype_cDNA_reverse      ------------------------------------------------------------   475
```

Alignment of knockout cDNA sequences using forward and reverse primers

```
NM_022290.2     = NCBI reference sequence
ORF             = Open reading frame
###             = Serine

NM_022290.2               CCGCCAGCGAGAAACTCTGGTCTTTAAAGGATGAAGAACTCAATGCTGGAGGCTGCGTCC   300
ORF                       ------------------------------ATGAAGAACTCAATGCTGGAGGCTGCGTCC   30
Knockout_cDNA_forward     ------------------------------ATGAAGAACAGT-TGCTGGAGGCTGCGTCC   28
Knockout_cDNA_reverse     ------------------------------ATGAAGAACAGT-TGCTGGAGGCTGCGTCC   29
                                                        *********### *****************

NM_022290.2               CTCCTTCTGGAGAAGCTGCTCCTTATTTCCAACTTCAAGATCTTTAGCGTGTGCGCCCCG   360
ORF                       CTCCTTCTGGAGAAGCTGCTCCTTATTTCCAACTTCAAGATCTTTAGCGTGTGCGCCCCG   90
Knockout_cDNA_forward     CTCCTTCTGGAGAAGCTGCTCCTTATTTCCAACTTCAAGATCTTTAGCGTGTGCGCCCCG   88
Knockout_cDNA_reverse     CTCCTTCTGGAGAAGCTGCTCCTTATTTCCAACTTCAAGATCTTTAGCGTGTGCGCCCCG   89
                          ************************************************************

NM_022290.2               GGAGGAGGCACAGGGAAGAAACATCCCTATGAAATCAACTCTTTTCTCCGGGGTGACGTG   420
ORF                       GGAGGAGGCACAGGGAAGAAACATCCCTATGAAATCAACTCTTTTCTCCGGGGTGACGTG   150
Knockout_cDNA_forward     GGAGGAGGCACAGGGAAGAAACATCCCTATGAAATCAACTCTTTTCTCCGGGGTGACGTG   148
Knockout_cDNA_reverse     GGAGGAGGCACAGGGAAGAAACATCCCTATGAAATCAACTCTTTTCTCCGGGGTGACGTG   149
                          ************************************************************

NM_022290.2               TTGGAAGTGTCACGGACCCATTTTACCCACTATGGGATCTACCTGGGGGACAACCGTGTC   480
ORF                       TTGGAAGTGTCACGGACCCATTTTACCCACTATGGGATCTACCTGGGGGACAACCGTGTC   210
Knockout_cDNA_forward     TTGGAAGTGTCACGGACCCATTTTACCCACTATGGGATCTACCTGGGGGACAACCGTGTC   208
Knockout_cDNA_reverse     TTGGAAGTGTCACGGACCCATTTTACCCACTATGGGATCTACCTGGGGGACAACCGTGTC   209
                          ************************************************************

NM_022290.2               GCCCATCTAATGCCTGACATCCTGTTGGCCCTGACCAGTGACAAGGAACGCACTCAGAAG   540
ORF                       GCCCATCTAATGCCTGACATCCTGTTGGCCCTGACCAGTGACAAGGAACGCACTCAGAAG   270
Knockout_cDNA_forward     GCCCATCTAATGCCTGACATCCTGTTGGCCCTGACCAGTGACAAGGAACGCACTCAGAAG   268
Knockout_cDNA_reverse     GCCCATCTAATGCCTGACATCCTGTTGGCCCTGACCAGTGACAAGGAACGCACTCAGAAG   269
                          ************************************************************

NM_022290.2               GTGGTCTCCAACAAGCGTCTCCTCCCAGGAGTCATTTGCAAGGTGGCCAGCATCCGTGTG   600
ORF                       GTGGTCTCCAACAAGCGTCTCCTCCCAGGAGTCATTTGCAAGGTGGCCAGCATCCGTGTG   330
Knockout_cDNA_forward     GTGGTCTCCAACAAGCGTCTCCTCCCAGGAGTCATTTGCAAGGTGGCCAGCATCCGTGTG   328
Knockout_cDNA_reverse     GTGGTCTCCAACAAGCGTCTCCTCCCAGGAGTCATTTGCAAGGTGGCCAGCATCCGTGTG   329
                          ************************************************************

NM_022290.2               GACACAGTAGAGGACTTTGCCTATGGAGCGGACATCCTCGTCAATCACCTAGACGAGACT   660
ORF                       GACACAGTAGAGGACTTTGCCTATGGAGCGGACATCCTCGTCAATCACCTAGACGAGACT   390
Knockout_cDNA_forward     GACACAGTAGAGGACTTTGCCTATGGAGCGGACATCCTCGTCAATCACCTAGACGAGACT   388
Knockout_cDNA_reverse     GACACAGTAGAGGACTTTGCCTATGGAGCGGACATCCTCGTCAATCACCTAGACGAGACT   389
                          ************************************************************

NM_022290.2               CTCAAGAAGAAGTCCTTGCTCAATGAGGAGGTGGCACGCAGAGCAGAGCAGCAGTTGGGG   720
ORF                       CTCAAGAAGAAGTCCTTGCTCAATGAGGAGGTGGCACGCAGAGCAGAGCAGCAGTTGGGG   450
Knockout_cDNA_forward     CTCAAGAAGAAGTCCTTGCTCAATGAGGAGGTGGCACGCAGAGCAGAGCAGCAGTTGGGG   448
Knockout_cDNA_reverse     CTCAAGAAGAAGTCCTTGCTCAATGAGGAGGTGGCACGCAGAGCAGAGCAGCAGTTGGGG   449
                          ************************************************************

NM_022290.2               CTGACCCCCTACAGCCTACTGTGGAACAACTGCGAACACTTTGTGACCTACTGCAGATAC   780
ORF                       CTGACCCCCTACAGCCTACTGTGGAACAACTGCGAACACTTTGTGACCTACTGCAGATAC   510
Knockout_cDNA_forward     CTGACCCCCTACAGCCTACTGTGGAACAACTGCGAACACTTTGTGACCTACTGCAGATAC   508
Knockout_cDNA_reverse     CTGACCCCCTACAGCCTACTGTGG------------------------------------   473
                          ************************

NM_022290.2               GGCTCTCCTATCAGTCCGCAGGCTGAGAAGTTTCACGAGACTGTGAAGATACTCATTCGT   840
ORF                       GGCTCTCCTATCAGTCCGCAGGCTGAGAAGTTTCACGAGACTGTGAAGATACTCATTCGT   570
Knockout_cDNA_forward     GGCTCTCCTATCAGTCCGCAGGCTGAGAAGTTTCACGAGACTGA----------------   552
Knockout_cDNA_reverse     ------------------------------------------------------------   473
```

Alignment of the wildtype human and rat Lrat ORF nucleotide sequences

```
LRAT_human      ATGAAGAACCCCATGCTGGAGGTGGTGTCTTTACTACTGGAGAAGCTGCTCCTCATCTCC      60
Lrat_rat        ATGAAGAACTCAATGCTGGAGGCTGCGTCCCTCCTTCTGGAGAAGCTGCTCCTTATTTCC      60
                ********* * **********  * ***  * ** ***************** ** ***

LRAT_human      AACTTCACGCTCTTTAGTTCGGGCGCCGCGGGCGAAGACAAAGGGAGGAACAGTTTTTAT      120
Lrat_rat        AACTTCAAGATCTTTAGCGTGTGCGCCCCGGGAGGAGGCACAGGGAAGAAACATCCCTAT      120
                ******* * *******    * ***** **** * ** ** ***** ***    *    ***

LRAT_human      GAAACCAGCTCTTTCCACCGAGGCGACGTGCTGGAGGTGCCCCGGACCCACCTGACCCAC      180
Lrat_rat        GAAATCAACTCTTTTCTCCGGGGTGACGTGTTGGAAGTGTCACGGACCCATTTTACCCAC      180
                **** ** ****** * *** ** ****** **** *** * ********  * ******

LRAT_human      TATGGCATCTACCTAGGAGACAACCGTGTTGCCCACATGATGCCCGACATCCTGTTGGCC      240
Lrat_rat        TATGGGATCTACCTGGGGGACAACCGTGTCGCCCATCTAATGCCTGACATCCTGTTGGCC      240
                ***** ******** ** *********** *****  * ***** ***************

LRAT_human      CTGACAGACGACATGGGGCGCACGCAGAAGGTGGTCTCCAACAAGCGTCTCATCCTGGGC      300
Lrat_rat        CTGACCAGTGACAAGGAACGCACTCAGAAGGTGGTCTCCAACAAGCGTCTCCTCCCAGGA      300
                *****    **** **  ***** *************************** ***  **

LRAT_human      GTTATTGTCAAAGTGGCCAGCATCCGCGTGGACACAGTGGAGGACTTCGCCTACGGAGCT      360
Lrat_rat        GTCATTTGCAAGGTGGCCAGCATCCGTGTGGACACAGTAGAGGACTTTGCCTATGGAGCG      360
                ** ***  *** ************** *********** ******** ***** *****

LRAT_human      AACATCCTGGTCAATCACCTGGACGAGTCCCTCCAGAAAAAGGCACTGCTCAACGAGGAG      420
Lrat_rat        GACATCCTCGTCAATCACCTAGACGAGACTCTCAAGAAGAAGTCCTTGCTCAATGAGGAG      420
                 ******* *********** ****** * *** **** *** *  ******* ******

LRAT_human      GTGGCGCGGAGGGCTGAAAAGCTGCTGGGCTTTACCCCCTACAGCCTGCTGTGGAACAAC      480
Lrat_rat        GTGGCACGCAGAGCAGAGCAGCAGTTGGGGCTGACCCCCTACAGCCTACTGTGGAACAAC      480
                ***** ** ** ** **  *** * ****  * ************** ***********

LRAT_human      TGCGAGCACTTCGTGACCTACTGCAGATATGGCACCCCGATCAGTCCCCAGTCCGACAAG      540
Lrat_rat        TGCGAACACTTTGTGACCTACTGCAGATACGGCTCTCCTATCAGTCCGCAGGCTGAGAAG      540
                ***** ***** **************** *** * ** ******** *** * ** ***

LRAT_human      TTTTGTGAGACTGTGAAGATAATTATTCGTGATCAGAGAAGTGTTCTTGCTTCAGCAGTC      600
Lrat_rat        TTTCACGAGACTGTGAAGATACTCATTCGTGATCAGAGAAGTTGTCTTGCTTCAGCTGTC      600
                ***    ************** * *****************  ************ ***

LRAT_human      TTGGGATTGGCGTCTATAGTCTGTACGGGCTTGGTATCATACACTACCCTTCCTGCAATT      660
Lrat_rat        TTGGGATTAGTGTCTATTATCTACACAGGCCTGGCATCATATATGACCCTTCCTGCAGTC      660
                ******** * ******  ***  ** *** *** ****** *  ************ *

LRAT_human      TTTATTCCATTCTTCCTATGGAT---GGCTGGCTAA                              693
Lrat_rat        TGCATCCCGTTCTGCTTGTGGATGATGTCTGGCTAG                              696
                *  ** ** **** * * *****   * *******
```

Alignment of the wildtype human and rat LRAT ORF amino acid sequences

```
*       = The same amino acid
:       = Indicates conservation between groups of strongly similar properties
.       = Indicates conservation between groups of weakly similar properties

LRAT_human      MKNPMLEVVSLLLEKLLLISNFTLFSSGAAGEDKGRNSFYETSSFHRGDVLEVPRTHLTH      60
Lrat_rat        MKNSMLEAASLLLEKLLLISNFKIFSVCAPGGGTGKKHPYEINSFLRGDVLEVSRTHFTH      60
                *** ***..*************.:**  * * ..*::  ** .** ******* ***:**

LRAT_human      YGIYLGDNRVAHMMPDILLALTDDMGRTQKVVSNKRLILGVIVKVASIRVDTVEDFAYGA      120
Lrat_rat        YGIYLGDNRVAHLMPDILLALTSDKERTQKVVSNKRLLPGVICKVASIRVDTVEDFAYGA      120
                ************:*********.*  ***********: *** *****************

LRAT_human      NILVNHLDESLQKKALLNEEVARRAEKLLGFTPYSLLWNNCEHFVTYCRYGTPISPQSDK      180
Lrat_rat        DILVNHLDETLKKKSLLNEEVARRAEQQLGLTPYSLLWNNCEHFVTYCRYGSPISPQAEK      180
                :********:*:**:***********: **:********************:*****::*

LRAT_human      FCETVKIIIRDQRSVLASAVLGLASIVCTGLVSYTTLPAIFIPFFLWMAG-               230
Lrat_rat        FHETVKILIRDQRSCLASAVLGLVSIIYTGLASYMTLPAVCIPFCLWMMSG               231
                * *****:****** ********.**: ***.** ****: *** *** .
```

Alignment of the mutant human and rat LRAT ORF nucleotide sequences

```
LRAT_human      ATGAAGAACCCATGCTGGAGGTGGTGTCTTTACTACTGGAGAAGCTGCTCCTCATCTCCA      60
Lrat_rat        ATGAAGAACTCATGCTGGAGGCTGCGTCCCTCCTTCTGGAGAAGCTGCTCCTTATTTCCA      60
                ********* ***********  * ***  * ** ***************** ** ****

LRAT_human      ACTTCACGCTCTTTAGTTCGGGCGCCGCGGGCGAAGACAAAGGGAGGAACAGTTTTTATG      120
Lrat_rat        ACTTCAAGATCTTTAGCGTGTGCGCCCCGGGAGGAGGCACAGGGAAGAAACATCCCTATG      120
                ****** * *******   * ***** **** * ** ** ***** ***   *   ****

LRAT_human      AAACCAGCTCTTTCCACCGAGGCGACGTGCTGGAGGTGCCCCGGACCCACCTGA------      174
Lrat_rat        AAATCAACTCTTTTCTCCGGGGTGACGTGTTGGAAGTGTCACGGACCCATTTTACCCACT      180
                *** ** ****** * *** ** ****** **** *** * ********  * *

LRAT_human      ---------------------------------------                           174
Lrat_rat        ATGGGATCTACCTGGGGGACAACCGTGTCGCCCATCTAA                           219
```

Alignment of the mutant human and rat LRAT theoretic ORF amino acid sequences

```
*       = The same amino acid
:       = Indicates conservation between groups of strongly similar properties
.       = Indicates conservation between groups of weakly similar properties

LRAT_human       MKNPCWRWCLYYWRSCSSSPTSRSLVRAPRAKTKGGTVFMKPALSTEATCWRCPGPT---     57
LRAT_rat         MKNSCWRLRPSFWRSCSLFPTSRSLACAPREEAQGRNIPMKSTLFSGVTCWKCHGPILPT     60
                 *** ***    :*****  ******. *** :::* .: ** :* : .***:* **

LRAT_human       ------------                                                     57
LRAT_rat         MGSTWGTTVSPI                                                     72
```
